# Prevalence of Hantaviruses Harbored by Murid Rodents in Northwestern Ukraine and Discovery of a Novel Puumala Virus Strain

**DOI:** 10.3390/v13081640

**Published:** 2021-08-18

**Authors:** Evan P. Williams, Mariah K. Taylor, Iryna Demchyshyna, Igor Nebogatkin, Olena Nesterova, Iryna Khuda, Lyudmyla Chernenko, Oleksandra A. Hluzd, Vira V. Kutseva, Gregory E. Glass, Nataliia Yanko, Colleen B. Jonsson

**Affiliations:** 1 Department of Microbiology, Immunology and Biochemistry, The University of Tennessee Health Science Center, Memphis, TN 38163, USA; ewilli99@uthsc.edu (E.P.W.); mtayl121@uthsc.edu (M.K.T.); 2 Department of Virology, State Institution Public Health Center of the Ministry of Health of Ukraine, 04071 Kyiv, Ukraine; i.demchyshyna@phc.org.ua (I.D.); l.chernenko@phc.org.ua (L.C.); o.hluzd@phc.org.ua (O.A.H.); v.kutseva@phc.org.ua (V.V.K.); 3 Public Health Center of the Ministry of Health of Ukraine, 01030 Kyiv, Ukraine; niv_zoo@ua.fm; 4 I.I. Schmalhausen Institute of Zoology of NAS of Ukraine, 01030 Kyiv, Ukraine; 5 Black and Veatch Special Projects Corp., 01033 Kyiv, Ukraine; nesterova.olena@gmail.com (O.N.); irina.khuda@gmail.com (I.K.); 6 Emerging Pathogens Institute and Department of Geography, University of Florida, Gainesville, FL 32610, USA; gglass@epi.ufl.edu; 7 Volyn Oblast Laboratory Center of the Ministry of Health of Ukraine, 43010 Lutsk, Ukraine; yankobsg@gmail.com

**Keywords:** *Puumala orthohantavirus*, Ukraine, next generation sequencing, field survey

## Abstract

In Europe, two species of hantaviruses, *Puumala orthohantavirus* (PUUV) and *Dobrava orthohantavirus* (DOBV), cause hemorrhagic fever with renal syndrome in humans. The rodent reservoirs for these viruses are common throughout Ukraine, and hence, the goal of this study was to identify the species and strains of hantaviruses circulating in this region. We conducted surveillance of small rodent populations in a rural region in northwestern Ukraine approximately 30 km from Poland. From the 424 small mammals captured, we identified nine species, of which the most abundant were *Myodes glareolus*, the bank vole (45%); *Apodemus flavicollis*, the yellow-necked mouse (29%); and *Apodemus agrarius*, the striped field mouse (14.6%) Using an indirect immunofluorescence assay, 15.7%, 20.5%, and 33.9% of the sera from *M. glareolus*, *A. glareolus*, and *A. flavicollis* were positive for hantaviral antibodies, respectively. Additionally, we detected antibodies to the hantaviral antigen in one *Microtus arvalis*, one *Mus musculus*, and one *Sorex minutus*. We screened the lung tissue for hantaviral RNA using next-generation sequencing and identified PUUV sequences in 25 small mammals, including 23 *M. glareolus*, 1 *M. musculus*, and 1 *A. flavicollis,* but we were unable to detect DOBV sequences in any of our *A. agrarius* specimens. The percent identity matrix and Bayesian phylogenetic analyses of the S-segment of PUUV from 14 *M. glareolus* lungs suggest the highest similarity (92–95% nucleotide or 99–100% amino acid) with the Latvian lineage. This new genetic information will contribute to future molecular surveillance of human cases in Ukraine.

## 1. Introduction

In the family *Hantaviridae*, order *Bunyavirales*, more than 47 distinct species of hantaviruses have been identified in rodents, moles, shrews, and bats across the globe [1]. Of these, some species within the genus *Orthohantavirus*, harbored by rodents, cause serious disease when transmitted to humans. In Europe and Asia, human cases of hemorrhagic fever with renal syndrome (HFRS) occur following aerosol transmission of excreta with hantaviruses carried by two rodent genera, *Apodemus* and *Myodes*. *Hantaan orthohantavirus* (HTNV) is carried by *Apodemus agrarius* and is found east of the Ural Mountains, in China, and Korea, while *Dobrava-Belgrade orthohantavirus* (DOBV) is harbored by *A. agrarius* (striped field mouse) and *A. flavicollis* (yellow-necked mouse) and is found in numerous European countries [2,3,4,5,6,7,8,9]. In Europe, *Puumala* *orthohantavirus* (PUUV) harbored by *Myodes glareolus* (bank vole) causes a less severe form of HFRS, which is referred to as nephropathia epidemica (NE) [10]. In some years, PUUV has caused up to 10,000 human cases in northern Europe and Russia. However, in contrast with HFRS, which has a case fatality rate (CFR) of up to 10%, NE disease has a lower CFR (<1%) [11].

**Figure 1:**
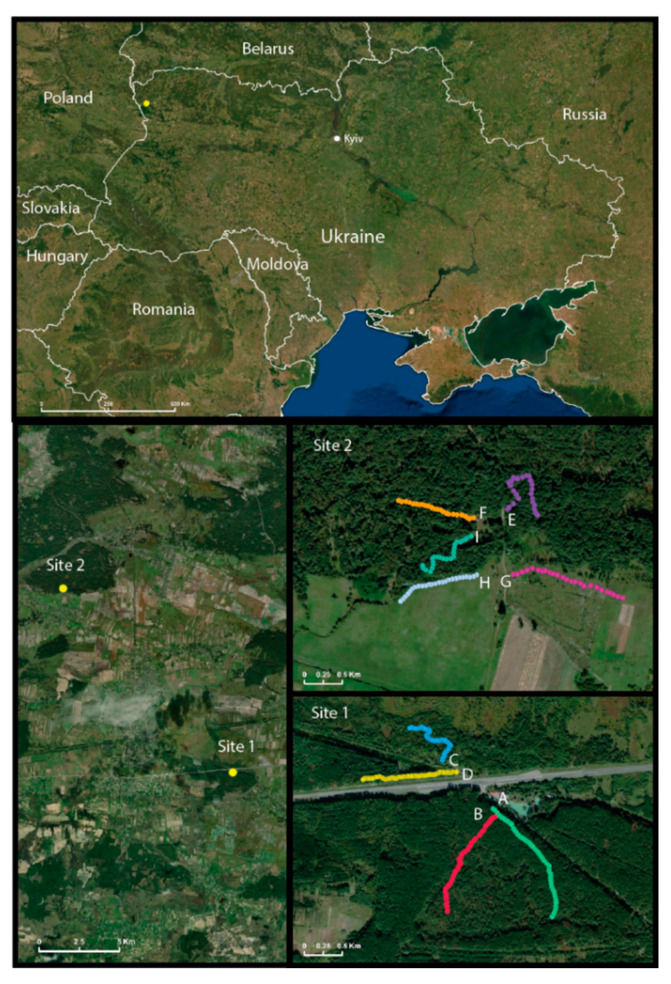
Survey location in northwestern Ukraine. The survey was conducted in northwestern Ukraine (**top**) at two distinct locations (**lower left** panel), which were ten km from each other: site 1, lines A–D and site 2, lines E–I (**lower right** panel).

Given the geographical location of Ukraine and prior surveillance of hantaviruses in humans and wildlife [12,13], we hypothesized that DOBV and PUUV, or even perhaps novel strains of hantaviruses, circulate in wild rodent populations in Ukraine. We previously reported the presence of antibodies to hantaviruses in *A. agrarius*, *A. flavicollis*, and *M. glareolus* in the western region of Ukraine [12]. In this prior study, however, we did not have specimens available for the genetic identification of the species circulating in Ukraine. We launched a field survey in September and October of 2019 of wild rodents in a rural region of Volyn Oblast, where we had detected antibodies to hantaviruses in rodents collected ten years prior (Figure 1).

The goal of this study was to identify the viral species and strains of hantaviruses circulating in western Ukraine. Toward this goal, we conducted a field survey of 424 captured small mammals in northwestern Ukraine in order to obtain and identify hantavirus species and the genotypes circulating in Ukraine. We report a high seroprevalence in our sampling region, 33.9% *A. agrarius*, 20.5% *A. flavicollis*, and 15.7% *M. glareolus*. Eight mammalian species were identified with antibodies to hantaviruses, of which we were able to sequence the virus from the lung tissue of three species. We designed primers for next-generation sequencing (NGS) and identified PUUV sequences from 23 specimens of *M. glareolus*. Additionally, we identified PUUV sequences within the lung tissue of two spillover hosts, *Mus musculus* and *A. flavicollis*. Bayesian phylogenetics support an association of PUUV sequences isolated from *M. glareolus* in Ukraine with the Latvian (LAT) lineage.

## 2. Materials and Methods

### 2.1. Small Mammal Collection

During two weeks in September and October 2019, 50 (Site 1, lines A–D) and 25 (Site 2, lines E–I) Sherman traps (7.6 × 8.9 × 22.9 cm, Sherman Trap Company, Tallahassee, FL, USA) were set, 10 m apart, along 500 or 250 m transects. Sites 1 and 2 were located 10 km apart from one another (Figure 1). These were staggered across nine collecting sites as effectively as possible (Table S1). We ran each line (except line H) for up to five nights, for a total of 167 line-nights, or 8350 trap-nights, with various success rates per line (Table S2).

Each small mammal was identified morphologically according to species and the weight; total body, tail, ear, and hind foot lengths were measured. Species were identified in the field. The sex and categorical age (juvenile or adult) were also recorded (Table S3). The lungs, heart, kidneys, muscles, and blood were harvested and stored immediately in liquid nitrogen, prior to transport to the Virology Department at the Public Health Center in Kyiv, where the samples were stored at −80 °C until processing.

### 2.2. Immunofluorescence Assay (IFA)

An indirect IFA was used to screen the blood samples for antibodies that cross-reacted with PUUV, HTNV, or DOBV grown in Vero E6 cells, as described previously [12,14,15]. Briefly, whole blood samples were diluted 1:10 in PBS in a 96-well plate and were incubated for 30 min at 37 °C on acetone-fixed, 10-spot slides. The slides were washed copiously in PBS. Twenty-microliters of a 1:500 dilution of Alexa Fluor 488 F (ab’)2 Fragment rabbit anti-mouse IgG (H+L) (Invitrogen, Waltham, MA, USA) was added and incubated for 30 min at 37 °C. The slides were washed copiously in PBS and each well was scored as positive or negative using a 20× objective with a Nikon epifluorescence microscope. Positive blood samples were titrated to the end-point by a two-fold serial dilution of blood from 1:32 through 1:8192, and were tested and scored microscopically for the characteristic punctate staining of hantaviruses.

### 2.3. Screening of Rodent Lung RNA by Reverse-Transcriptase Polymerase Chain Reaction (RT-PCR) for PUUV and DOBV

The total RNA was isolated from all of the available rodent lung specimens following homogenization in TRIzol Reagent (Invitrogen) with a Bead Mill 4 (Fisher Scientific, Waltham, MA, USA), and was resuspended in 25–30 µL nuclease-free water. Nested PCR was used to confirm the presence of viral nucleic acids in each lung tissue specimen. The first step of the nested PCR was performed with SuperScript IV One-Step RT-PCR System (Invitrogen) with 40 cycles and a 60 °C annealing temperature, as well as PUUV or DOBV vRNA-specific primers amplifying a 1kb region of the S- or M-segment following the manufacturer’s protocols (Table S4). For the second nested PCR step, 2.5 µL of the first reaction’s product was used with a Platinum SuperFi PCR Master Mix (Invitrogen) and PUUV or DOBV vRNA-specific primers, amplifying approximately a 500 bp region of the S- or M-segment with 30–35 PCR cycles (Table S4). PCR reactions were visualized on the E-Gel SizeSelect II Agarose Gel 2% (Invitrogen) using the E-Gel Power Snap Electrophoresis System (Invitrogen).

### 2.4. Next-Generation Sequencing

For NGS, the total RNA was quantitated on the Qubit 4 Fluorometer (Invitrogen) using the Qubit RNA HS Assay Kit (Invitrogen). cDNAs were synthesized from up to 1 µg total RNA from each lung sample, using a mixture of primers that amplified the vRNA or cRNA of each segment (Table S4) with SuperScript IV First-Strand Synthesis System (Invitrogen), following the manufacturer’s protocol. The three genomic segments were amplified with Platinum SuperFi PCR Master Mix (Invitrogen) with 35 cycles at a 60 °C annealing temperature following the manufacturer’s protocols, along with a disjointed primer pooling approach in which three PCR reactions produced non-overlapping amplicons, which, when combined, would completely cover the genome twice [16]. For each specimen, the PCR products of each segment were combined separately based on whether they were synthesized from vRNA or cRNA, and were purified using the Wizard SV Gel and PCR Clean-Up System (Promega, Madison, WI, USA), with the final purified PCR product being eluted in 15 µL nuclease-free water. Sequencing libraries were created using Nextera XT DNA Library Preparation Kit (Illumina, San Diego, CA, USA) according to the manufacturer’s guidelines, with the exception that the input concentration was 1 ng/µL, tagmentation time was extended to 10 min, and the final volume of each library was in 15 µL of resuspension buffer. Two final libraries, each containing 40 libraries, were pooled into a final concentration of 4.2 nM with a 1% PhiX spike-in and were sequenced on a MiSeq using two MiSeq Reagent Kits v3 150 cycles (Illumina). Sequencing reads were demultiplexed and transferred onto the CLC Genomics Workbench v.21.0.2 (Qiagen, Hilden, Germany) to analyze the sequencing reads. The reads were quality trimmed and mapped to the reference genome of each of the S-, M-, and L-segments of our lab strain PUUV strain K27 (Genbank accession no. MZ673552-MZ673554) using a length fraction of 0.8 and a similarity fraction of 0.5, with the other parameters set to default. The consensus sequence was extracted with a low coverage threshold set to 1, incorporating a quality score requirement to resolve conflicts within each base position. To further our understanding of our genome amplification approach, we created a detailed mapping report to identify the percent genome coverage, number of aligned reads, and average depth of coverage by excluding short reads (<15 nt) or long reads (>1500 nt). All of the vRNA sequences were deposited at GenBank (accession no. MZ673485-MZ673551).

### 2.5. Phylogenetic Analyses

We performed MUSCLE alignments using a 765 nt sequence of each S-segment, as well as three representative sequences from each PUUV lineage—Alp-Adrian (ALAD), Central European (CE), Danish (DAN), Finnish (FIN), LAT, North-Scandinavian (N-SCA), South-Scandinavian (S-SCA), and Russian (RUS) [17]. The MUSCLE alignment of the 633 nt M-segment sequence included PUUV sequences from Bosnia and Herzegovina, Croatia, Germany, Finland, France, Lithuania, Russia, and Sweden, and a 542 nt alignment of the L-segment included PUUV sequences from Germany, Finland, France, Lithuania, Russia, and Sweden. We additionally included five DOBV sequences as an outgroup for the S-, M-, and L-segment alignments. We examined the relationships through maximum likelihood using MEGA X [18]. Phylogenetic trees were constructed using PhyML 3.0 on the ATGC server (http://www.atgc-montpellier.fr/phyml/, accessed on 23 July 2021), using a maximum likelihood method and general time reversible model with default settings, with the exception of 1000 bootstrap replicates, as well as having altered the proportion of invariable sites (0.247, 0.219, and 0.224) and gamma shape parameters (0.299, 0.322, and 0.354) for each S-, M-, and L-alignment, respectively [19,20]. The MUSCLE alignments were also used to create a percent identity matrix (PIM) using Clustal2.1 (https://www.ebi.ac.uk/Tools/msa/clustalo/, accessed on 24 June 2021) using default parameters [21,22].

### 2.6. Statistical Analyses

Descriptive statistics were used to describe the distribution of the captured rodents and the prevalence of hantavirus infection using GraphPad Prism 9 v.9.1 (GraphPad Software, San Diego, CA, USA). We analyzed the prevalence of hantaviruses detected by hantaviral Ab/RNA with sex, age, and weight as the independent variables using the Fishers Exact Test to calculate the *p*-value and the odds ratio and its confidence interval.

### 2.7. Ethics Statements

All of the animal procedures were approved (ACUP No. 18–108, date of approval 4 June 2019) by the UTHSC Institutional Animal Care and Use Committee (IACUC), which follows the eighth Edition of the Guide for the Care and Use of Laboratory Animals (Guide), NRC 2011, and the Animal Care and Use Committee guidelines of the American Society of Mammologists for the use of wild mammals in research and education. The study did not involve endangered or protected species. The study was conducted as part of the routine rodent surveillance program of the Ministry of Health.

## 3. Results

### 3.1. Distribution of Small Mammal Species

A total of 424 small mammals were captured along nine distinct lines (A–I) where Sherman trap lines were set at 10 m apart. Lines A–C were set in a forest and line D was set adjacent to a four-lane roadway in shrubs; the roadway separated lines A and B from lines C and D (Figure 1). Lines E, F, and I were placed in a forest, and lines G and H were placed in shrubs and grasslands near the forest’s edge nearby small agricultural farms (Figure 1). The collection was completed over two weeks in the last week of September and the first week of October 2019, and over that period, we captured small mammals representing seven genera and nine species (Table 1). These included species from the orders, Carnivora, Eulipotyphla, and Rodentia. Line D had the majority of captures with 120 animals (28%; 95%CI: 24.22–32.77%), followed by line C with 92 animals (22%; 95%CI: 18–25%). Only one rodent was captured from line H during the first night of trapping; therefore, this line was moved to another field site and was omitted from diversity studies, where mentioned. *M. glareolus* (45%; 95%CI: 40.38–49.81%) was the most captured small mammal in total, followed by *A. flavicollis* (29%; 95%CI: 24.89–33.50%) and then *A. agrarius* (15%; 95%CI: 11.58–18.30%).

### 3.2. Evidence and Distribution of Antibodies to Hantavirus by Line

The capture lines varied among each other based on the number of captured animals, as well as the number of hantavirus-positive animals (Table 1 and Table 2). Molecular diagnostics showed that 79 of the animals tested positive for the presence of hantavirus by serology. Of the small mammals that were captured and tested by IFA for the presence of hantavirus antibodies, only mammals from the orders of Eulipotyphla and Rodentia tested positive. Antibody-positive animals were captured on every line except for line B. The most hantavirus seropositive animals were captured on line C with 23 animals (29%; 95%CI: 20–40%), followed by line D with 16 animals (20%; 95%CI: 13–30%). There was no difference between the number of captured male and female animals that were positive for hantavirus antibodies (odds ratio: 1.087; 95%CI: 0.6475–1.819; *p* = 0.7905). Adults (83%) made up the largest age group of small mammals captured, with only 73 (17%) being juvenile (data not shown). The distribution of the endpoint reciprocal titer of the antibody-positive animals is presented in Table 3. IFA reciprocal titers showed a vast distribution ranging from 1:32 to 1:8192, indicating that recent reservoir infections are occurring. In addition to the *Myodes* and *Apodemus* species, we identified one *Sorex minutus*, one *Microtus arvalis*, and one *Mus musculus* that were antibody positive. Of these, only the *Mus musculus* was advanced for NGS because of tissue availability.

### 3.3. Rodent Diversity within Each Line

The Shannon index was calculated for each line as a measure of the species diversity (Table 4). Line A had the highest Shannon index value (1.46) and had a proportion of hantavirus (HV) of 0.2, which was the average for all of the lines, and line D had the second highest Shannon index value (1.44), but had the lowest proportion of HV at 0.1, and was also the line where most small mammals were captured. There was no relationship found between the Shannon index and the number of HV positive animals (R^2^ = 0.25) or the proportion of HV positive animals (R^2^ = 0.31).

### 3.4. Association of Population Structure and Prevalence of Antibody to Hantavirus in Myodes glareolus and Apodemus Species

The distribution of the presence of antibodies to hantavirus was stratified by categorical age and sex (Table 5). Among the three most abundantly captured rodents, neither sex nor age was associated with the rodents being hantaviral positive. We noted a similar level of antibody in juvenile and adults in both male *Apodemus* species. This trend did not hold in the females, as the number of juveniles captured was much lower and so no comparative results could be drawn. Overall, a similar level of antibody presence was measured in male or female adults within each species.

**Figure 2:**
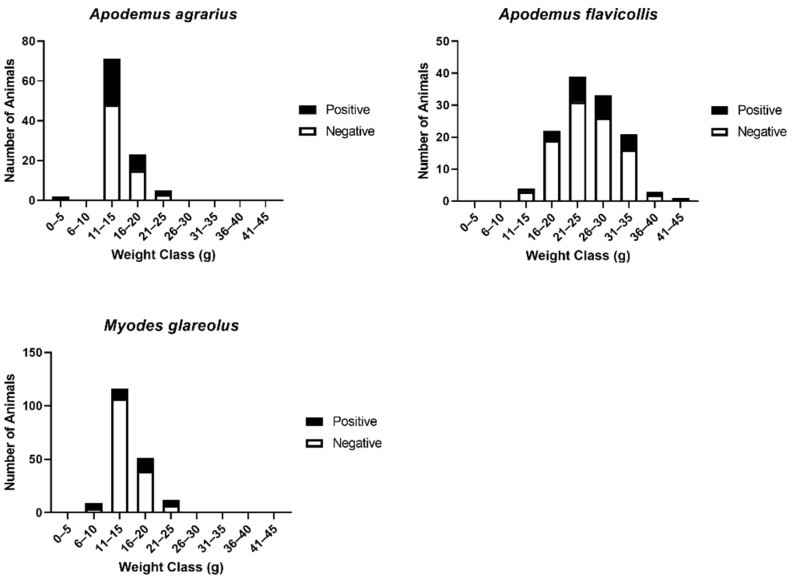
Weight range of rodents and percent hantavirus for *A. agrarius*, *A. flavicollis,* and *M. glareolus*.

The weight class of the three small mammal species that had the greatest number of animals that were hantavirus seropositive were 11–15 g for *A. agrarius,* 21–25 g for *A. flavicollis,* and 16–20 g for the *M. glareolus* (Figure 2). This weight range suggests that most of these were subadults or adults.

### 3.5. Next-Generation Sequencing and Phylogenetic Analyses of Viral Sequences Amplified from Myodes glareolus Lung

To determine the sequence of the hantavirus genotypes in antibody-positive mice, we developed an amplicon-based approach to obtain full length sequences for L-, M-, and S-segments based on the genomes of PUUV and DOBV. The amplicons were designed to overlap by 500 bp across the genome; however, all of the primers could not be multiplexed without certain regions losing depth of coverage. This was likely due to primer bias and primer dimer issues. Hence, we identified three pools of primers that would generate equivocal levels of amplicons in a two-step RT-PCR [16]. The total RNA was extracted from the lungs of animals identified as antibody positive. As might be expected in animal tissues, full length consensus sequences from each specimen were not obtained, except for the S-segment. Moreover, despite the efficiency of the approach for DOBV purified from seed stocks (similar to PUUV), we were unable to obtain useful information to identify the DOBV carried by these tissues.

PUUV sequences were recovered from 25 total rodents—23 *M. glareolus*, 1 *M. musculus*, and 1 *A.* *flavicollis*. Because of the low genome coverage of the genomic RNA of PUUV from the S-, M-, and L-genomes of *M. musculus* (47%, 23%, and 10%, respectively) and *A.* *flavicollis* (18%, 8%, and 4%, respectively), additional analyses were not performed (data not shown). We found that there was no difference between the percent genome coverage collected from obtaining vRNA compared with cRNA for the S- and M-segments, while the L-segment favored vRNA.

**Figure 3:**
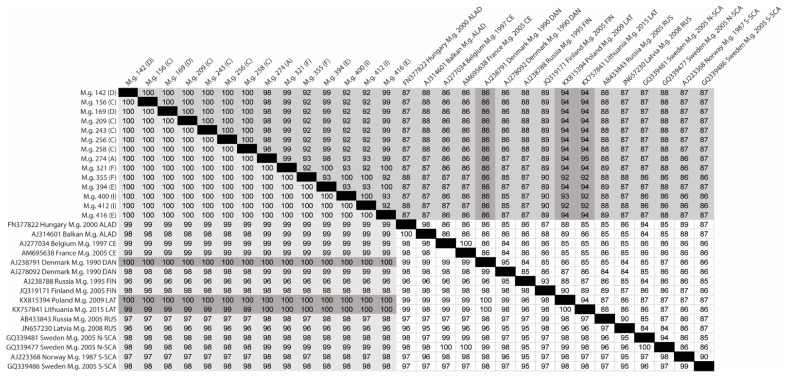
Percent identity matrix of PUUV S-segment cRNA of Ukrainian isolates and representative sequences of the eight PUUV lineages. Nucleotide similarity is reported above the black diagonal, and amino acid similarity is reported below the black diagonal. Light grey highlight represents the Ukraine isolates’ amino acid similarity; medium grey highlight represents the Ukraine isolates’ nucleotide similarity; and dark grey highlight represents the LAT lineage similarity with the Ukraine isolates and the DAN sequence, which had 100% amino acid similarity but 86% nucleotide similarity. Each virus isolate is listed by the GenBank accession number, the country the isolate was collected from, the host species (*Myodes glareolus* (M.g)), the year collected, and its associated PUUV lineage. Ukraine isolates are listed according to the host species, specimen number, and capture line.

PUUV sequences were obtained from 23 *M. glareolus* by NGS, and were aligned and analyzed using rapid sequence distance estimation. Fourteen PUUV sequences were aligned to two sequences within each PUUV lineage to generate a percent identity matrix (PIM) of a 722 nt region (genome position 43–764 nt) of the S-segment cRNA (Figure 3). This analysis showed a strong association of the Ukraine isolates with the LAT lineage sharing a 92–95% nucleotide identity and 99–100% amino acid identity. Interestingly, we identified one sequence within the DAN lineage (GenBank accession no. AJ238791) that shared a 100% amino acid identity to every Ukraine isolate, similar to what was observed in the LAT lineage, while the nucleotide identity was only 86%. Analyses of the N protein sequence revealed two amino acid sites that were unique to our genomes in comparison with the other lineages assessed in our dataset, N168K aa/T507A nt and Q428H aa/A1284T nt (Table S5). Additionally, a GnGc protein alignment with 62 PUUV CDS sequences provided information about four amino acid markers that set our Ukraine isolates apart from others (Q/R/I/M57K, G/T/S78A, S185A, and N/T244S). Three of the four amino acids were shared with a sequence obtained from *M. glareolus* from Lithuania, which is likely part of the LAT lineage. Within the RNA-dependent RNA polymerase (RdRp), we identified two unique amino acid markers S761G and D2140E. Three other unique markers in RdRp were shared with a sequence isolated from *M. glareolus* collected from Lithuania in 2015 (GenBank accession no. MT514294), L759F, Q/R/H777L, and S1646A.

**Figure 4:**
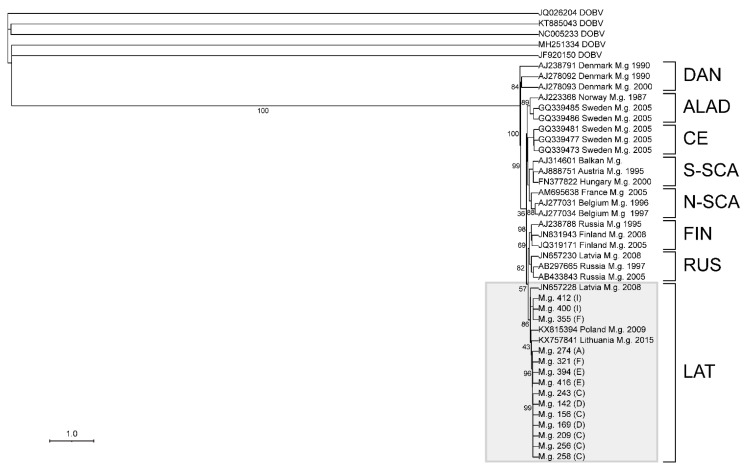
Phylogenetic tree of the partial PUUV S-segment. The tree was generated from an alignment to a 765 nt sequence at position 1–765 nt/1–241 aa (genome position is noted from reference sequence NC_005224). Branch support values for each major node are displayed. The Latvian (LAT) lineage is highlighted within the grey box, and the line from which each Ukraine isolate was collected is shown in parenthesis. Each virus isolate is listed by GenBank accession number, the country the isolate was collected from, the host species (*Myodes glareolus* (M.g)), and the year collected. The eight PUUV lineages are listed to the right of the tree.

**Figure 5:**
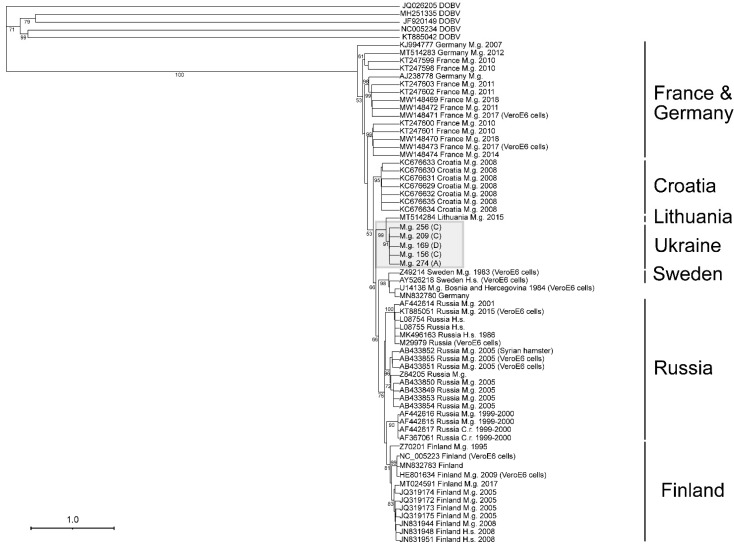
Phylogenetic tree of a partial PUUV M-segment. The tree was generated from an alignment of a 633nt sequence at position 2190–2823 nt/717–928 aa (genome position is noted from reference sequence NC_005223). Branch support values for each major node are displayed. Ukraine isolates are highlighted within the grey box, and the line from which each Ukraine isolate was collected is shown in parenthesis. Each virus isolate is listed according to the GenBank accession number, the country the isolate was collected from, the host species (*Myodes glareolus* (M.g.), *Homo sapiens* (H.s.), and *Clethrionomys rufocanus* (C.r.)), the year collected, and whether the isolate was sequenced directly from the host or amplified in another host (cell lines or Syrian hamster).

**Figure 6:**
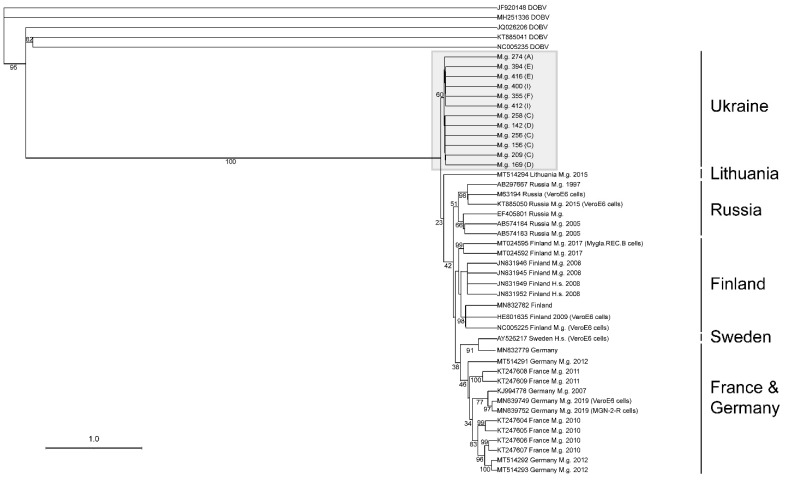
Phylogenetic tree of a partial PUUV L-segment. The tree was generated from an alignment of a 542 nt sequence at position 2278–2820 nt/748–927 aa (genome position is noted from reference sequence NC_005225). Branch support values for each major node are displayed. Ukraine isolates are highlighted within the grey box, and the line from which each Ukraine isolate was collected is shown in parenthesis. Each virus isolate is listed according to GenBank accession number, the country the isolate was collected from, the host species (*Myodes glareolus* (M.g)), the year collected, and whether the isolate was sequenced directly from the host or amplified in another host (cell lines or Syrian hamster).

An S-segment alignment, using maximum likelihood on the PhyML 3.0 on the ATGC server, of 14 sequences containing 765 nt, provided sufficient information for the assessment of the lineage that the viral sequence is associated with (Figure 4). As there are not sufficient PUUV M- or L-segment sequences, the confirmation of the lineage was not possible (Figure 5 and Figure 6).

As shown in Figure 4, the S-segment showed a strong association with the Latvian (LAT) lineage. This lineage includes viruses from Latvia, Lithuania, and Poland, and our sequences suggests that western Ukraine should be included in this lineage [23]. A 633 nt alignment of 58 M-segment PUUV sequences showed that Ukraine isolates have a strong association with those captured from *M. glareolus* in Lithuania (Figure 5). In contrast, a 542 nt alignment of the L-segment did not cluster the Ukraine sequences with other published PUUV L sequences (Figure 6).

The S-segment sequence phylogeny and geographical coordinates show four groups of isolates from *M. glareolus* collected from neighboring lines (Figure 4). Specimen IDs 355, 400, and 412 formed the first group, which were identified in neighboring lines I and F. Specimen ID 274 was found in line A, which was physically separated from the other lines with a highway. Specimen IDs 321, 394, and 416 were collected from neighboring lines E and F. Similarly, IDs 142, 156, 169, 209, 243, 256, and 258 were sequenced from rodents collected on lines C and D. The L-segment phylogeny supports these groupings as it also maintains the separation between these four groupings. As the M-segment phylogeny only had two groups represented, there was not enough evidence to support this separation.

## 4. Discussion

The prevalence of HFRS cases in humans remains unknown for Ukraine, although recent research reported a 1.6% seroprevalence of the hantaviral antibody in 966 healthy individuals within a small locality in Lviv Oblast, Ukraine [13]. The lack of information on diseases caused by hantaviruses is two-fold; firstly, there have been few surveillance efforts on the reservoirs of viruses that cause these diseases, and secondly, there is lack of molecular tools for the diagnostic identification of the causative virus strains in the region. The prevalence of reservoir hosts of hantaviruses and the incidence of cases in neighboring countries suggests that hantaviruses are circulating within the country, although there has only been one previous study that reported the seroprevalence within humans and the rodent reservoir [12,13]. Our previous work studying antibody prevalence in Volyn during spring and autumn reported a seroprevalence of 2.2% (9/404) in *A. agrarius* and 7% (31/433) in *M. glareolus*. Our current 2019 survey conducted in autumn showed a seroprevalence of 33.9% (21/62) in *A. agrarius*, 20.5% (25/122) in *A. flavicollis*, and 15.7% (30/191) in *M. glareolus*. With this study, we show that for people living in western Ukraine, there is a high potential for HFRS caused by PUUV. Our results will enable the development of qRT-PCR assays to confirm diagnosis in febrile patients with symptoms suggestive of the spectrum of illness caused by hantaviruses.

Apart from our previous study that suggested hantaviruses are present in Ukraine, little is known about the species or associated disease. All of the countries surrounding Ukraine (Belarus, Hungary, Moldova, Poland, Russia, Romania, and Slovakia) have reported cases of HFRS, but the species or genotype of the viruses that caused the disease or the reservoir of the virus is not known in some cases [24,25,26,27,28,29]. Most hantavirus identifications reported in these studies have focused on humans.

Only a small number of field trapping surveys have been conducted in surrounding countries. In a large field survey spanning multiple districts southwest of where this study was conducted in the Subcarpathia region in Poland, 194 rodents were captured, with the most abundantly captured rodents consisting of *A. agrarius*, *A. flavicollis,* and *M. glareolus,* as was found in this study [30]. From the 194 captured rodents, 17 showed evidence of hantavirus infection by PCR and/or IFA for DOBV, PUUV, and TULV. In this study, the percentage of positive rodents was half of the current study. In another field survey in western Poland, which captured 106 small mammals, the most abundantly captured rodents (*A. agrarius*, *A. flavicollis,* and *M. glareolus*) were the same as in our study, but only five of the animals tested positive for RNA, and these animals tested positive for DOBV and *Seewis orthohantavirus* (SWSV) [31]. We not only identified PUUV in *M. glareolus,* but we also identified PUUV sequences within the reservoir host of DOBV. Our study shows that reservoir hosts can act as spillover hosts to harbor other species of hantaviruses. Lastly, in northeast Poland, a field survey was conducted that led to the first reported isolation of PUUV in Poland, where three captured rodents out of 45 tested positive for PUUV [32]. In summary, Ukraine and the region would benefit from additional survey studies that include the molecular identification of the variants.

Similar to what has been observed in other research, we observed 100% amino acid identity of the PUUV S-segment fragments to the LAT lineage within our trapping sites [33]. Despite other PUUV lineages, we observed no distinct molecular signatures within our S-segments, suggesting that the Ukrainian sequences are not a new lineage. As previously described by Razzauti et al. [23], who collected two groups of *M. glareolus* in Latvia, which were distinguished by PUUV sequences within either the RUS and LAT lineages, we identified all 11 synapomorphic amino acid markers in the N protein sequence in our sequences containing the positions specific to *M. glareolus* found within the LAT lineage (A5T, R30K, V34M, Y61F, P233A, D234E, K237R, D250E, R265K, N272Q, and D302N), which strongly supports our suggestion to add our Ukraine isolates to the LAT lineage. Similar to Razzuti et al. [23], we confirmed eight of nine synaptomorphic markers in the M protein sequence Y769F, T771S, V803A, V807I, R810K, V815A, T827S, and I/L835V. We also confirmed five of eight RdRp synaptomorphic amino acid markers, R200K, Q234P, V239A, N242S, and Q265H. In contrast with Razzauti et al. [23], we had two sequences containing D245 rather than S245 in the RdRp. Due to regions of low sequence coverage, we could not confirm the other two markers, D273E or R315Q. In distinguishing unique markers that separate Ukraine within that of its LAT lineage, we identified several markers within the N protein N168K aa/T507A nt and Q428H aa/A1284T nt, within the GnGc protein Q/R/I/M57K, and within the RdRp protein S761G and D2140E. Future work will focus on uncovering the complete CDS of Ukraine isolates to confirm all lineage-specific synaptomorphic markers within the S-, M-, and L-segments.

In agreement with Castel et al. [34] following the phylogeography of PUUV in eastern Europe, our PUUV isolates confirm a close relationship with isolates from Poland and Lithuania. Following the dispersion route of PUUV throughout eastern Europe, Castel [34] shows a potential route from southwestern Ukraine up towards Lithuania, suggesting that the “Eastern” refugia expansion may characterize Ukraine isolates as being ancestral to those found in northeastern Europe. In addition, the article by Razzauti et al. [23], which identified the PUUV LAT lineage, found that *M. glareolus* mtDNA originated from the Carpathian clade. They suggested that other members of the LAT lineage would likely be identified in Poland, Lithuania, and Ukraine, among others, if *M. glareolus* from the Carpathian clade carrying PUUV from the LAT lineage were in Latvia after the Last Glacial Maximum [23]. From our S-segment phylogenetic analyses (Figure 4), we show that Ukraine isolates cluster together with a Poland isolate collected in 2009 and a Lithuanian isolate collected in 2015. As the Carpathian Mountains separates Ukraine from southwestern Europe, the limited geographic barriers to North and Eastern Europe may have allowed for easier access for PUUV dispersal through the East European Plain. The addition of our work in adding PUUV isolates from Ukraine to the current database of PUUV isolates across Europe and Asia will aid in the phylogeographic analyses to uncover how these lineages have spread across the Old World.

This study describes the circulation of PUUV in multiple rodent species in Ukraine, which is likely an important driver of HFRS cases. The study further shows that PUUV circulating in northwestern Ukraine is genetically related to the PUUV found in the LAT lineage. This work emphasizes the necessity of field surveys to identify viruses that will allow for the development of more accurate molecular diagnostic tools for suspected HFRS cases, and to provide a further understanding of the impact these viruses have on human health.

## Figures and Tables

**Table 1:** Distribution of captured small mammal species by line.

Species	Capture Lines									Total
	**A**	**B**	**C**	**D**	**E**	**F**	**G**	**H**	**I**	
*Apodemus agrarius*	4	0	11	20	3	0	21	1	2	**62**
*Apodemus flavicollis*	19	16	18	42	7	13	2	0	6	**123**
*Microtus arvalis*	0	0	0	2	0	0	3	0	1	**6**
*Microtus subterraneus*	1	0	0	1	0	0	0	0	0	**2**
*Mus musculus*	1	0	1	0	0	0	0	0	0	**2**
*Muscardinus avellanarius*	1	0	0	0	0	0	0	0	0	**1**
*Mustela nivales*	0	0	1	1	0	0	0	0	0	**2**
*Myodes glareolus*	9	24	51	39	24	25	2	0	17	**191**
*Sorex minutus*	5	0	10	15	3	1	1	0	0	**35**
**Total**	**40**	**40**	**92**	**120**	**37**	**39**	**29**	**1**	**26**	**424**

**Table 2:** Distribution of antibody-positive small mammal species by line.

Species	Capture Lines									Total
	**A**	**B**	**C**	**D**	**E**	**F**	**G**	**H**	**I**	
*Apodemus agrarius*	2 (4)	0	3 (11)	3 (20)	1 (3)	0	11 (21)	1 (1)	2	**21**
*Apodemus flavicollis*	3 (19)	0	5 (18)	8 (42)	3 (7)	4 (13)	0	0	2 (6)	**25**
*Microtus arvalis*	0	0	0	1 (2)	0	0	0	0	0	**1**
*Microtus subterraneus*	1	0	0	1	0	0	0	0	0	**0**
*Mus musculus*	1 (1)	0	1	0	0	0	0	0	0	**1**
*Muscardinus avellanarius*	1	0	0	0	0	0	0	0	0	**0**
*Mustela nivales*	0	0	1	1	0	0	0	0	0	**0**
*Myodes glareolus*	2 (9)	0	14 (51)	4 (39)	5 (24)	3 (25)	0	0	2 (17)	**30**
*Sorex minutus*	0	0	1 (10)	0	0	0	0	0	0	**1**
**Total**	**8**	**0**	**23**	**16**	**9**	**7**	**11**	**1**	**4**	**79**

**Table 3:** Distribution of IFA reciprocal titers in rodent reservoir species of PUUV and DOBV.

Species	IFA Reciprocal Titers								
	**1:32**	**1:64**	**1:128**	**1:256**	**1:512**	**1:1024**	**1:2048**	**1:4096**	**1:8192**
*Apodemus agrarius*	0	5	5	3	1	3	1	2	1
*Apodemus flavicollis*	1	6	5	1	7	4	1	1	1
*Microtus arvalis*	0	0	0	0	1	0	0	0	0
*Mus musculus*	0	0	0	0	1	0	0	0	0
*Myodes glareolus*	0	1	7	9	2	4	3	2	3
*Sorex minutus*	0	1	0	0	0	0	0	0	0

**Table 4:** Overall Hantaviral Ab status and Shannon diversity in captured rodents by line.

Line	A	B	C	D	E	F	G	I
**HV-Negative**	32	40	69	104	28	32	18	22
**HV-Positive**	8	0	23	16	9	7	11	4
**Total No. Rodents**	40	40	92	120	37	39	29	26
**Proportion of HV**	0.2	0.0	0.3	0.1	0.2	0.2	0.4	0.2
**SHANNON (H)**	1.46	0.67	1.24	1.44	1.00	0.75	0.95	0.94

**Table 5:** *A. agrarius*, *A. flavicollis,* and *M. glareolus* captured according to age, sex, and Ab prevalence.

	*A. agrarius*			*A. flavicollis*			*M. glareolus*		
	Total No.	Total No. Ab Pos *	% Pos	Total No.	Total No. Ab Pos	% Pos	Total No.	Total No. Ab Pos	% Pos
**MALE**									
Juvenile	3	1	33.3	22	4	18.2	35	6	16.7
Adult	31	11	35.5	60	14	23.3	108	16	15.0
**FEMALE**									
Juvenile	2	0	0	7	1	14.3	1	0	0
Adult	26	9	34.6	33	6	18.2	47	8	17
**Total**	**62**	**21**		**122**	**25**		**191**	**30**	****

* Ab Pos: Antibody Positives.

## Data Availability

All supporting data to the manuscript are hosted in Zenodo http://doi.org/10.5281/zenodo.5089927 (accessed on 31 July 2021).

## Supplementary Materials

The following are available online at https://www.mdpi.com/article/10.3390/v13081640/s1, Table S1: Trap coordinates, Table S2: Line and day success rate, Table S3: Small mammal database, Table S4: Primers used to amplify DOBV or PUUV hantaviruses. DOBV primers were designed based on an alignment of sequences from Russia, Germany, Slovenia, Slovakia, Croatia, Estonia, Greece, Finland from *Apodemus agrarius* and *A. flavicollis* rodents and *Homo sapiens* hosts. PUUV primers were designed based on alignments from sequences collected from Russia, Germany, Slovenia, Slovakia, Finland, France, Czech Republic. The optimal annealing temperature for these primers is set at 60 °C, Table S5: NGS depth of coverage of conserved S-, M-, L-segment sites in Ukraine isolates from sequenced vRNA.
