# Supplementary material for: Prevalence of Hantaviruses Harbored by Murid Rodents in Northwestern Ukraine and Discovery of a Novel Puumala Virus Strain
Source: Viruses. 2021 Aug 18;13(8):1640. doi: 10.3390/v13081640 (PMC8402871; doi:10.3390/v13081640)
Supplement: Supplementary file 1 [file viruses-13-01640-s001.zip › TableS1 Trap Coordinates.pdf]

Table S1. Trap Coordinates

ID	Trap numb	Latitude	Longitude	Elevation
1	A0001	51.20326	24.08906	155.9159
2	A0002	51.20321	24.08916	154.143
3	A0003	51.20312	24.08931	155.244
4	A0004	51.20301	24.08946	155.8412
5	A0005	51.20293	24.0896	154.4603
6	A0006	51.2029	24.0897	154.2177
7	A0007	51.20284	24.08979	155.7012
8	A0008	51.2028	24.08993	157.0822
9	A0009	51.20274	24.09006	156.9516
10	A0010	51.20267	24.09018	155.4586
11	A0011	51.20252	24.09029	156.4757
12	A0012	51.20246	24.09035	156.681
13	A0013	51.20239	24.09045	155.8599
14	A0014	51.20229	24.09056	157.4274
15	A0015	51.20222	24.09069	156.5877
16	A0016	51.20217	24.09081	157.6794
17	A0017	51.20214	24.09089	158.0899
18	A0018	51.20207	24.09105	158.3885
19	A0019	51.20201	24.0912	156.9516
20	A0020	51.20198	24.09125	156.9516
21	A0021	51.2019	24.09135	156.9516
22	A0022	51.20183	24.09147	156.9516
23	A0023	51.20173	24.09161	156.9516
24	A0024	51.20166	24.09175	156.9516
25	A0025	51.20157	24.09183	156.9516
26	A0026	51.20147	24.09194	158.6871
27	A0027	51.20139	24.09205	158.827
28	A0028	51.20132	24.09211	158.5751
29	A0029	51.20127	24.09217	158.4072
30	A0030	51.20118	24.09231	158.3232
31	A0031	51.20109	24.09243	157.7913
32	A0032	51.20097	24.09243	157.4274
33	A0033	51.20091	24.09243	157.698
34	A0034	51.20082	24.0925	157.67
35	A0035	51.2007	24.09252	157.4181
36	A0036	51.20063	24.09252	157.6887
37	A0037	51.20053	24.09253	157.1662
38	A0038	51.20041	24.09255	154.6282
39	A0039	51.20029	24.09259	155.9998
40	A0040	51.20018	24.09267	156.7743
41	A0041	51.2001	24.09274	154.115
42	A0042	51.19999	24.09276	155.8505
43	A0043	51.19991	24.09279	156.0185
44	A0044	51.19979	24.09283	154.7682
45	A0045	51.19968	24.09285	155.3187

46	A0046	51.19954	24.09282	153.1913
47	A0047	51.19945	24.09281	154.283
48	A0048	51.19936	24.09277	154.6469
49	A0049	51.19927	24.09271	155.2161
50	A0050	51.19916	24.09261	152.9394
51	B0001	51.20296	24.08916	158.5098
52	B0002	51.20294	24.08908	158.5098
53	B0003	51.2029	24.08899	158.5098
54	B0004	51.20283	24.08891	158.5098
55	B0005	51.20275	24.08882	158.5098
56	B0006	51.20269	24.08872	158.5098
57	B0007	51.20261	24.08861	158.5098
58	B0008	51.20247	24.08851	158.5098
59	B0009	51.20243	24.08844	158.5098
60	B0010	51.20241	24.08839	159.1723
61	B0011	51.20228	24.08825	160.1613
62	B0012	51.20214	24.088	161.225
63	B0013	51.20209	24.08792	161.225
64	B0014	51.20198	24.08776	161.225
65	B0015	51.20189	24.0877	161.225
66	B0016	51.20183	24.08762	161.225
67	B0017	51.20174	24.08752	161.225
68	B0018	51.20165	24.08737	161.225
69	B0019	51.2016	24.08724	161.2157
70	B0020	51.20153	24.08716	161.2157
71	B0021	51.20143	24.0871	155.8599
72	B0022	51.20137	24.08701	156.9236
73	B0023	51.20128	24.08707	158.1272
74	B0024	51.20119	24.08712	158.4725
75	B0025	51.20113	24.08704	158.4725
76	B0026	51.20106	24.08697	158.4725
77	B0027	51.20101	24.08694	158.4725
78	B0028	51.20096	24.08688	158.4725
79	B0029	51.20091	24.08681	158.4725
80	B0030	51.20087	24.08678	158.4725
81	B0031	51.2008	24.08672	158.4725
82	B0032	51.20069	24.08666	154.1803
83	B0033	51.20057	24.0866	156.3451
84	B0034	51.20051	24.08655	154.6469
85	B0035	51.20044	24.08649	153.6485
86	B0036	51.20037	24.08641	154.8615
87	B0037	51.20033	24.08636	154.4043
88	B0038	51.20027	24.08626	155.1974
89	B0039	51.20022	24.0862	154.8522
90	B0040	51.20012	24.08619	154.143
91	B0041	51.20006	24.08621	155.2534
92	B0042	51.19997	24.08624	154.4603

93	B0043	51.1999	24.08623	154.4976
94	B0044	51.1998	24.08625	154.7589
95	B0045	51.19972	24.08629	156.1864
96	B0046	51.1997	24.08627	156.0838
97	B0047	51.19964	24.08625	155.5706
98	B0048	51.19953	24.08628	155.7106
99	B0049	51.19942	24.08632	202.9052
100	B0050	51.19936	24.08631	204.6687
101	C0001	51.20509	24.08601	199.6675
102	C0002	51.20516	24.08603	199.6675
103	C0003	51.2052	24.08607	199.6675
104	C0004	51.20526	24.08613	199.6675
105	C0005	51.20533	24.08612	199.6675
106	C0006	51.20535	24.08616	199.6675
107	C0007	51.20541	24.08622	199.6675
108	C0008	51.20547	24.08632	199.6675
109	C0009	51.20551	24.08637	199.145
110	C0010	51.20555	24.0864	199.2289
111	C0011	51.20562	24.08644	199.4529
112	C0012	51.20564	24.08651	199.0423
113	C0013	51.20569	24.08645	198.9863
114	C0014	51.20573	24.08638	198.6877
115	C0015	51.20578	24.0863	198.5758
116	C0016	51.20579	24.08624	198.5758
117	C0017	51.20585	24.08618	198.5758
118	C0018	51.20583	24.08613	198.5758
119	C0019	51.20585	24.08602	198.5758
120	C0020	51.20585	24.08592	198.5758
121	C0021	51.20584	24.08587	198.5758
122	C0022	51.20582	24.08579	198.5758
123	C0023	51.20583	24.08573	198.5758
124	C0024	51.20584	24.08567	198.5758
125	C0025	51.20582	24.08555	198.5758
126	C0026	51.20584	24.08542	198.5758
127	C0027	51.20586	24.08532	198.5758
128	C0028	51.2059	24.0852	198.5758
129	C0029	51.20592	24.08514	198.5758
130	C0030	51.20594	24.08511	198.5758
131	C0031	51.20599	24.08508	198.5758
132	C0032	51.20606	24.08508	198.5758
133	C0033	51.20609	24.08513	198.5758
134	C0034	51.20612	24.08516	198.5758
135	C0035	51.20614	24.0852	198.5758
136	C0036	51.20619	24.08523	198.5758
137	C0037	51.20624	24.08518	198.5758
138	C0038	51.20624	24.08509	198.5758
139	C0039	51.20626	24.08503	197.8387

140	C0040	51.20627	24.08495	198.1839
141	C0041	51.20631	24.08486	198.5758
142	C0042	51.20634	24.08473	198.3145
143	C0043	51.20631	24.08465	197.68
144	C0044	51.20629	24.08458	197.68
145	C0045	51.20628	24.08453	197.68
146	C0046	51.20628	24.08443	197.68
147	C0047	51.20628	24.08438	197.68
148	C0048	51.2063	24.08429	200.1153
149	C0049	51.20632	24.08414	198.2959
150	C0050	51.2063	24.08405	201.319
151	D0001	51.20464	24.08685	195.0954
152	D0002	51.20464	24.08685	184.9609
153	D0003	51.20465	24.08661	195.8512
154	D0004	51.20462	24.08652	195.8512
155	D0005	51.20462	24.08642	195.8512
156	D0006	51.20464	24.08634	195.8512
157	D0007	51.20463	24.08622	195.8512
158	D0008	51.20463	24.08611	195.8512
159	D0009	51.20464	24.08603	195.8512
160	D0010	51.20464	24.08589	195.8512
161	D0011	51.20464	24.08583	195.8512
162	D0012	51.2046	24.08575	195.8512
163	D0013	51.20457	24.08562	195.8512
164	D0014	51.20455	24.08552	195.8512
165	D0015	51.20455	24.08536	195.8512
166	D0016	51.20456	24.08525	195.8512
167	D0017	51.20454	24.08514	195.8512
168	D0018	51.20452	24.08499	195.8512
169	D0019	51.20451	24.08488	195.8512
170	D0020	51.20452	24.08476	196.2991
171	D0021	51.2045	24.08465	196.0192
172	D0022	51.2045	24.08454	195.8979
173	D0023	51.2045	24.08437	191.951
174	D0024	51.20449	24.08421	191.951
175	D0025	51.20449	24.08412	191.951
176	D0026	51.20449	24.08394	191.951
177	D0027	51.20447	24.08375	191.923
178	D0028	51.20447	24.08365	194.1997
179	D0029	51.20445	24.08352	196.2898
180	D0030	51.20445	24.08338	196.4857
181	D0031	51.20441	24.08322	195.0768
182	D0032	51.20441	24.0831	196.7283
183	D0033	51.20444	24.08296	196.7563
184	D0034	51.20441	24.08284	196.8869
185	D0035	51.20439	24.08272	196.5697
186	D0036	51.20438	24.08259	196.6817

187	D0037	51.20438	24.08252	196.4297
188	D0038	51.20441	24.08245	194.6569
189	D0039	51.20443	24.08237	196.9616
190	D0040	51.20446	24.08228	196.8589
191	D0041	51.20447	24.08221	197.6894
192	D0042	51.20446	24.08211	197.5867
193	D0043	51.20445	24.08201	197.5681
194	D0044	51.20441	24.08183	197.6147
195	D0045	51.20441	24.08176	198.1466
196	D0046	51.20441	24.08162	198.1746
197	D0047	51.2044	24.08151	198.3052
198	D0048	51.2044	24.08138	198.3985
199	D0049	51.2044	24.0813	197.5494
200	D0050	51.20436	24.08123	198.7437
201	E0001	51.26836	23.99378	183.7307
202	E0002	51.2684	23.99385	181.7805
203	E0003	51.26849	23.99401	181.6312
204	E0004	51.26862	23.99421	182.1351
205	E0005	51.26872	23.99413	182.3124
206	E0006	51.2688	23.99402	182.5083
207	E0007	51.26893	23.9939	182.2097
208	E0008	51.269	23.99383	182.2471
209	E0009	51.26904	23.99394	181.0901
210	E0010	51.26912	23.99401	181.454
211	E0011	51.2691	23.99412	180.9594
212	E0012	51.2691	23.99417	181.2393
213	E0013	51.26914	23.99436	180.9781
214	E0014	51.26917	23.99451	180.7075
215	E0015	51.26916	23.99462	181.1927
216	E0016	51.26905	23.99467	179.5598
217	E0017	51.26895	23.99473	181.1087
218	E0018	51.26885	23.99473	180.5769
219	E0019	51.26873	23.9947	180.8288
220	E0020	51.26862	23.99474	180.7448
221	E0021	51.26853	23.99476	180.2596
222	E0022	51.26845	23.99483	181.3047
223	E0023	51.26835	23.99482	180.9314
224	E0024	51.26826	23.99488	181.5006
225	E0025	51.26821	23.99494	182.2191
226	F0001	51.26815	23.99247	182.555
227	F0002	51.26815	23.99236	181.1834
228	F0003	51.26813	23.9923	179.8304
229	F0004	51.26816	23.99223	181.1181
230	F0005	51.26819	23.99214	181.23
231	F0006	51.26822	23.99203	180.8288
232	F0007	51.26822	23.99183	180.6049
233	F0008	51.26823	23.99169	181.4446

234	F0009	51.26824	23.99151	181.314
235	F0010	51.26826	23.99142	182.0885
236	F0011	51.2683	23.9913	181.5099
237	F0012	51.26834	23.9912	181.2207
238	F0013	51.26836	23.99107	181.0154
239	F0014	51.26837	23.99097	181.5566
240	F0015	51.2684	23.99086	181.5659
241	F0016	51.26844	23.99072	181.5379
242	F0017	51.26845	23.99059	180.8568
243	F0018	51.26847	23.99048	179.1026
244	F0019	51.26847	23.99037	181.7619
245	F0020	51.26849	23.99026	179.6438
246	F0021	51.26849	23.99014	179.8118
247	F0022	51.26852	23.99001	180.2503
248	F0023	51.26855	23.98987	180.0077
249	F0024	51.26855	23.98969	181.7712
250	F0025	51.26857	23.98958	180.8195
251	G0001	51.26678	23.994	180.0077
252	G0002	51.26679	23.99416	180.0077
253	G0003	51.26684	23.99435	180.0077
254	G0004	51.26689	23.99454	180.0077
255	G0005	51.26694	23.99475	180.0077
256	G0006	51.26689	23.99497	180.0077
257	G0007	51.26686	23.99513	180.0077
258	G0008	51.26687	23.99523	180.0077
259	G0009	51.26683	23.99543	182.3217
260	G0010	51.2668	23.99554	182.1724
261	G0011	51.26677	23.99571	182.583
262	G0012	51.26671	23.99592	182.8536
263	G0013	51.26666	23.99606	183.2921
264	G0014	51.26661	23.99626	183.3761
265	G0015	51.26661	23.9964	183.7307
266	G0016	51.26657	23.9966	183.8986
267	G0017	51.26651	23.99698	184.1879
268	G0018	51.26647	23.99723	184.1225
269	G0019	51.26641	23.99741	184.1319
270	G0020	51.26636	23.99763	184.7664
271	G0021	51.26634	23.99775	185.1489
272	G0022	51.26629	23.99788	185.4008
273	G0023	51.26625	23.99804	185.1302
274	G0024	51.26622	23.99824	185.0276
275	G0025	51.2665	23.99673	185.0276
276	H0001	51.26677	23.9926	180.8568
277	H0002	51.26674	23.99247	180.8568
278	H0003	51.26672	23.99233	180.8568
279	H0004	51.26673	23.99219	180.8568
280	H0005	51.26672	23.99206	180.8568

281	H0006	51.26671	23.99193	180.8568
282	H0007	51.26669	23.9918	180.8568
283	H0008	51.26667	23.99166	180.8568
284	H0009	51.26665	23.99152	180.8568
285	H0010	51.26661	23.99138	180.8568
286	H0011	51.2666	23.99127	175.6689
287	H0012	51.26659	23.99114	180.8848
288	H0013	51.26656	23.99099	179.9797
289	H0014	51.26655	23.99085	180.6795
290	H0015	51.26654	23.99074	180.1383
291	H0016	51.26651	23.99063	179.5318
292	H0017	51.26651	23.9905	179.1679
293	H0018	51.2665	23.99034	179.3639
294	H0019	51.26648	23.9902	178.7574
295	H0020	51.26643	23.9901	179.0466
296	H0021	51.26637	23.99002	179.1959
297	H0022	51.26631	23.98994	179.3546
298	H0023	51.26624	23.98983	177.843
299	H0024	51.26619	23.98975	177.8243
300	H0025	51.26614	23.98966	178.7947
301	I0001	51.26771	23.99239	218.4222
302	I0002	51.26766	23.99222	220.8761
303	I0003	51.26763	23.99215	220.8761
304	I0004	51.2676	23.99204	220.8761
305	I0005	51.26757	23.99196	220.8761
306	I0006	51.26757	23.99183	220.8761
307	I0007	51.26751	23.99175	220.8761
308	I0008	51.26743	23.99173	220.8761
309	I0009	51.26735	23.99171	221.2214
310	I0010	51.26729	23.99171	220.4189
311	I0011	51.26721	23.99171	222.0891
312	I0012	51.26716	23.9916	220.2043
313	I0013	51.26716	23.99142	219.3646
314	I0014	51.26717	23.9913	220.4563
315	I0015	51.2672	23.99117	220.4563
316	I0016	51.2672	23.99105	220.4563
317	I0017	51.2672	23.99094	220.4563
318	I0018	51.26716	23.99089	220.4563
319	I0019	51.26708	23.99091	220.4563
320	I0020	51.26701	23.99092	218.8887
321	I0021	51.26694	23.99088	220.7268
322	I0022	51.26685	23.99081	220.2603
323	I0023	51.2669	23.99063	219.1406
324	I0024	51.26694	23.99053	219.3366
325	I0025	51.267	23.99051	220.0644
