# Supplementary material for: Prevalence of Hantaviruses Harbored by Murid Rodents in Northwestern Ukraine and Discovery of a Novel Puumala Virus Strain
Source: Viruses. 2021 Aug 18;13(8):1640. doi: 10.3390/v13081640 (PMC8402871; doi:10.3390/v13081640)
Supplement: Supplementary file 1 [file viruses-13-01640-s001.zip › TableS2 Line success rate.pdf]

Table S2. Line and day success rate

Line	N of traps	Date of collection	Morning or afternoon collection	Animals caugt (data sheets)	Total per day (animals, data sheets)	Alive (field collection team)	Day N for a trap
A	50	24-Sep	morning	8	9	no data	1
A	42	24-Sep	afternoon	1		no data	1
A	50	25-Sep	morning	5	6	no data	2
A	45	25-Sep	afternoon	1		no data	2
A	50	26-Sep	morning	9	24	no data	3
A	41	26-Sep	afternoon	15		no data	3
B	39	24-Sep	morning	12	14	no data	1
B	27	24-Sep	afternoon	2		no data	1
B	48	25-Sep	morning	8	12	no data	2
B	40	25-Sep	afternoon	4		no data	2
B	50	26-Sep	morning	15	15	no data	3
B	35	26-Sep	afternoon	0		N/A	3
C	50	25-Sep	morning	19	25	no data	1
C	31	25-Sep	afternoon	6		no data	1
C	50	26-Sep	morning	19	19	no data	2
C	31	26-Sep	afternoon	0		N/A	2
C	50	29-Sep	morning	18	18	8	3
C	32	29-Sep	afternoon	0		N/A	3
C	50	30-Sep	morning	15	15	6	4
C	35	30-Sep	afternoon	0		N/A	4
C	50	1-Oct	morning	6	6	4	5
C	44	1-Oct	afternoon				5
D	50	25-Sep	morning	29	32	no data	1
D	21	25-Sep	afternoon	3		no data	1
D	50	26-Sep	morning	26	26	no data	2
D	24	26-Sep	afternoon	0		N/A	2
D	50	29-Sep	morning	20	20	15	3
D	30	29-Sep	afternoon	0		N/A	3
D	50	30-Sep	morning	20	20	13	4
D	30	30-Sep	afternoon	0		N/A	4
D	50	1-Oct	morning	11		4	5
D	39	1-Oct	afternoon				5
E	25	29-Sep	morning	13	13	10	1
E	12	29-Sep	afternoon	0		N/A	1
E	25	30-Sep	morning	8	8	6	2
E	17	30-Sep	afternoon	0		N/A	2
E	25	1-Oct	morning	9		5	3
E	16	1-Oct	afternoon				3
F	25	29-Sep	morning	14	15	12	1
F	11	29-Sep	afternoon	1		1	1

F	25	30-Sep	morning	9	9		6	2
F	16	30-Sep	afternoon	0		N/A		2
F	25	1-Oct	morning	8			5	3
F	17	1-Oct	afternoon					3
G	25	29-Sep	morning	6	7		3	1
G	19	29-Sep	afternoon	1			0	1
G	25	30-Sep	morning	10	10		3	2
G	15	30-Sep	afternoon	0		N/A		2
G	25	1-Oct	morning	5			2	3
G	20	1-Oct	afternoon					3
H(a)	25	29-Sep	morning	1	2	N/A		1
H(a)	24	29-Sep	afternoon	1			1	1
H(b)	25	30-Sep	morning	12	12		11	1
H(b)	13	30-Sep	afternoon	0		N/A		1
H(b)	25	1-Oct	morning	8			6	2
H(b)	17	1-Oct	afternoon					2
				**378**			**121**	

Success rate morning / afternoon	Success rate per day	Comment
16.0%	18.0%	
2.4%	N/A	
10.0%	12.0%	
2.2%	N/A	
18.0%	48.0%	
36.6%	N/A	
30.8%	35.9%	
7.4%	N/A	
16.7%	25.0%	
10.0%	N/A	
30.0%	30.0%	
0.0%	N/A	
38.0%	50.0%	
19.4%	N/A	
38.0%	38.0%	
0.0%	N/A	
36.0%	36.0%	
0.0%	N/A	
30.0%	30.0%	
0.0%	N/A	
12.0%		
58.0%	64.0%	
14.3%	N/A	
52.0%	52.0%	
0.0%	N/A	
40.0%	40.0%	
0.0%	N/A	
40.0%	40.0%	
0.0%	N/A	
22.0%		
52.0%	52.0%	
0.0%	N/A	
32.0%	32.0%	
0.0%	N/A	
36.0%		
56.0%	60.0%	
9.1%	N/A	

36.0%	36.0%	
0.0%	N/A	
32.0%		
24.0%	28.0%	
5.3%	N/A	
40.0%	40.0%	
0.0%	N/A	
20.0%		
4.0%	8.0%	total from field: 0 (No. 343)
4.2%	N/A	
48.0%	48.0%	
0.0%	N/A	
32.0%		
		total in data sheets: 379
