# Supplementary material for: Prevalence of Hantaviruses Harbored by Murid Rodents in Northwestern Ukraine and Discovery of a Novel Puumala Virus Strain
Source: Viruses. 2021 Aug 18;13(8):1640. doi: 10.3390/v13081640 (PMC8402871; doi:10.3390/v13081640)
Supplement: Supplementary file 1 [file viruses-13-01640-s001.zip › TableS3 Small mammal database Ukraine.pdf]

Table S3. Small mammal database

1_Specime	2_Ab_or_F	3_Date	4_Trap_Lir	5_Trap_Nu	6_Status	7_Weight	8_Genus	9_Species	10_Male_c	11_Age	12_Blood	13_Heart	14_Lung	15_Kidney	16_Muscle	17_Total_E	18_Tail_Le	19_Hind_fc	20_Ear_ler	21_Time_c	22_IFA_1::	23_IFA_1::	24_IFA_SC	25_RTPCR_	26_Comments
248	0	26/09/2019	B	12	Dead	16.5	Apodemus	flavicolus	Male	juvenile	No	Yes	Yes	Yes	Yes	754	750	221	168	Am	NEGATIVE	NOT TESTE	0	Not Tested	
312	0	29/09/2019	C	36	Dead	7.6	Sorex	minutus	Female	adult	No	Yes	Yes	Yes	Yes	679	357	121	52	Am	NEGATIVE	NOT TESTE	0	Not Tested	
523	1	2/10/2019	F	17	Dead	23	Apodemus	flavicolus	Male	adult	No	Yes	Yes	Yes	Yes	936	1010	260	194	Am	POSITIVE	POSITIVE	64	NEGATIVE	
522	0	2/10/2019	F	6	Dead	7.8	Sorex	minutus	Male	adult	No	Yes	Yes	Yes	Yes	690	403	135	74	Am	NEGATIVE	NOT TESTE	0	Not Tested	
521	0	2/10/2019	F	5	Dead	11.5	Myodes	glareolus	Male	juvenile	No	Yes	Yes	Yes	Yes	729	357	174	144	Am	NEGATIVE	NOT TESTE	0	Not Tested	
520	0	2/10/2019	F	7	Dead	16.1	Myodes	glareolus	Male	adult	No	Yes	Yes	Yes	Yes	786	405	160	130	Am	NEGATIVE	NOT TESTE	0	Not Tested	
519	0	2/10/2019	I	13	Dead	15.1	Myodes	glareolus	Male	adult	No	Yes	Yes	Yes	Yes	847	360	184	150	Am	NEGATIVE	NOT TESTE	0	Not Tested	
518	0	2/10/2019	F	13	Dead	13.7	Myodes	glareolus	Male	adult	No	Yes	Yes	Yes	Yes	744	414	180	142	Am	NEGATIVE	NOT TESTE	0	Not Tested	
517	0	2/10/2019	F	21	Dead	16.3	Myodes	glareolus	Male	adult	No	Yes	Yes	Yes	Yes	800	391	173	105	Am	NEGATIVE	NOT TESTE	0	Not Tested	
516	0	2/10/2019	I	10	Dead	17.6	Microtus	arvalis	Female	adult	No	Yes	Yes	Yes	Yes	900	446	117	142	Am	NEGATIVE	NOT TESTE	0		
515	0	2/10/2019	G	8	Dead	7.3	Sorex	minutus	Male	adult	No	Yes	Yes	Yes	Yes	635	405	140	84	Am	NEGATIVE	NOT TESTE	0	Not Tested	
514	0	2/10/2019	E	17	Dead	7.2	Sorex	minutus	Male	adult	No	Yes	Yes	Yes	Yes	650	374	142	63	Am	NEGATIVE	NOT TESTE	0	Not Tested	
513	0	2/10/2019	G	4	Dead	14.1	Apodemus	agrarius	Female	adult	No	Yes	Yes	Yes	Yes	767	651	203	110	Am	NEGATIVE	NOT TESTE	0	Not Tested	
512	0	2/10/2019	G	5	Dead	16.9	Apodemus	agrarius	Female	adult	No	Yes	Yes	Yes	Yes	885	724	207	140	Am	NEGATIVE	NOT TESTE	0	Not Testec	Fleas
511	0	2/10/2019	G	21	Alive	13.2	Microtus	arvalis	Male	adult	Yes	Yes	Yes	Yes	Yes	834	311	179	118	Am	NEGATIVE	NOT TESTE	0	Not Tested	
510	0	2/10/2019	G	11	Alive	13.6	Microtus	arvalis	Male	adult	Yes	Yes	Yes	Yes	Yes	857	310	160	132	Am	NEGATIVE	NOT TESTE	0	Not Tested	
509	0	2/10/2019	G	19	Alive	24.7	Apodemus	flavicolus	Male	adult	Yes	Yes	Yes	Yes	Yes	1019	1010	265	192	Am	NEGATIVE	NOT TESTE	0	Not Tested	
508	0	2/10/2019	E	8	Dead	8.3	Sorex	minutus	Female	adult	No	Yes	Yes	Yes	Yes	603	420	134	75	Am	NEGATIVE	NOT TESTED		Not Tested	
507	0	2/10/2019	I	9	Alive	23.3	Apodemus	agrarius	Male	adult	Yes	Yes	Yes	Yes	Yes	1004	1000	250	170	Am	NEGATIVE	NOT TESTE	0	Not Tested	
506	0	2/10/2019	I	8	Dead	14.2	Myodes	glareolus	Male	adult	Yes	Yes	Yes	Yes	Yes	772	382	183	117	Am	NEGATIVE	NOT TESTE	0	Not Testec	Small amount of blood taken
505	0	2/10/2019	I	18	Alive	30.8	Apodemus	flavicolus	Female	adult	Yes	Yes	Yes	Yes	Yes	1115	1077	262	212	Am	NEGATIVE	NOT TESTE	0	Not Testec	Lactating
504	0	2/10/2019	E	9	Alive	15.1	Myodes	glareolus	Male	adult	Yes	Yes	Yes	Yes	Yes	870	363	173	115	Am	NEGATIVE	NOT TESTE	0	Not Tested	
503	0	2/10/2019	E	20	Alive	15.5	Myodes	glareolus	Female	adult	Yes	Yes	Yes	Yes	Yes	866	445	162	137	Am	NEGATIVE	NOT TESTE	0	Not Tested	
502	0	2/10/2019	E	14	Alive	15.2	Myodes	glareolus	Female	adult	Yes	Yes	Yes	Yes	Yes	885	406	165	143	Am	NEGATIVE	NOT TESTE	0	Not Tested	
501	0	2/10/2019	I	11	Alive	19.8	Apodemus	flavicolus	Male	adult	Yes	Yes	Yes	Yes	Yes	907	973	245	172	Am	NEGATIVE	NOT TESTE	0	Not Testec	Moribund, fleas
500	0	2/10/2019	E	11	Alive	15.6	Myodes	glareolus	Female	adult	Yes	Yes	Yes	Yes	Yes	883	384	165	143	Am	NEGATIVE	NOT TESTE	0	Not Tested	
499	0	2/10/2019	E	13	Alive	17.8	Myodes	glareolus	Male	adult	Yes	Yes	Yes	Yes	Yes	874	346	186	122	Am	NEGATIVE	NOT TESTE	0	Not Tested	
498	0	2/10/2019	D	28	Dead	13.9	Apodemus	agrarius	Male	adult	No	Yes	Yes	Yes	Yes	744	644	190	109	Am	NEGATIVE	NOT TESTE	0	Not Tested	
497	0	2/10/2019	C	18	Dead	8.5	Sorex	minutus	Male	adult	No	Yes	Yes	Yes	Yes	596	408	142	55	Am	NEGATIVE	NOT TESTE	0	Not Tested	
496	0	2/10/2019	D	39	Alive	14.6	Myodes	glareolus	Female	adult	Yes	Yes	Yes	Yes	Yes	900	356	174	142	Am	NEGATIVE	NOT TESTE	0	Not Testec	Lactating
495	1	2/10/2019	C	11	Dead	8	Sorex	minutus	Male	adult	No	Yes	Yes	Yes	Yes	706	349	146	54	Am	POSITIVE	POSITIVE	64	Not Tested	
494	0	2/10/2019	C	26	Dead	11.1	Myodes	glareolus	Male	juvenile	No	Yes	Yes	Yes	Yes	695	353	170	109	Am	NEGATIVE	NOT TESTE	0	Not Tested	
493	0	2/10/2019	D	8	Alive	13.5	Apodemus	agrarius	Male	adult	Yes	Yes	Yes	Yes	Yes	803	662	193	100	Am	NEGATIVE	NOT TESTE	0	Not Testec	Moribund
492	0	2/10/2019	C	9	Dead	12.6	Myodes	glareolus	Female	adult	No	Yes	Yes	Yes	Yes	763	445	190	120	Am	NEGATIVE	NOT TESTE	0	Not Tested	
491	0	2/10/2019	C	37	Dead	13.8	Apodemus	agrarius	Female	adult	No	Yes	Yes	Yes	Yes	798	662	190	108	Am	NEGATIVE	NOT TESTE	0	Not Tested	
490	0	2/10/2019	D	23	Alive	20.5	Apodemus	flavicolus	Male	juvenile	Yes	Yes	Yes	Yes	Yes	895	819	227	172	Am	NEGATIVE	NOT TESTE	0	Not Tested	
489	0	2/10/2019	D	31	Alive	20.5	Apodemus	flavicolus	Male	adult	Yes	Yes	Yes	Yes	Yes	928	914	238	174	Am	NEGATIVE	NOT TESTE	0	Not Tested	
488	0	2/10/2019	D	40	Alive	25.4	Apodemus	flavicolus	Female	adult	Yes	Yes	Yes	Yes	Yes	988	814	240	172	Am	NEGATIVE	NOT TESTE	0	Not Tested	
487	0	2/10/2019	C	23	Alive	16.8	Apodemus	flavicolus	Male	juvenile	Yes	Yes	Yes	Yes	Yes	878	874	247	158	Am	NEGATIVE	NOT TESTE	0	Not Tested	
486	0	2/10/2019	C	34	Alive	14.2	Myodes	glareolus	Male	adult	Yes	Yes	Yes	Yes	Yes	869	367	168	126	Am	POSITIVE	NEGATIVE	0	Not Tested	
485	0	2/10/2019	D	1	Alive	11.6	Microtus	subterrane	Male	adult	Yes	Yes	Yes	Yes	Yes	790	226	138	86	Am	NEGATIVE	NOT TESTE	0	Not Testec	Microtus subterraneus
484	0	2/10/2019	C	8	Alive	18.9	Apodemus	flavicolus	Male	adult	Yes	Yes	Yes	Yes	Yes	914	753	226	154	Am	NEGATIVE	NOT TESTE	0	Not Tested	
483	0	2/10/2019	D	32	Alive	13.8	Myodes	glareolus	Female	adult	Yes	Yes	Yes	Yes	Yes	901	368	169	125	Am	NEGATIVE	NOT TESTE	0	Not Testec	Fleas
482	0	2/10/2019	D	47	Alive	19	Apodemus	agrarius	Female	adult	Yes	Yes	Yes	Yes	Yes	915	626	200	110	Am	NEGATIVE	NOT TESTE	0	Not Tested	
481	0	2/10/2019	C	19	Alive	6.1	Sorex	minutus	Female	adult	Yes	Yes	Yes	Yes	Yes	621	355	111	59	Am	NEGATIVE	NOT TESTE	0	Not Tested	
480	0	2/10/2019	D	50	Dead	8.2	Myodes	glareolus	Male	juvenile	Yes	Yes	Yes	Yes	Yes	663	349	164	108	Am	NEGATIVE	NOT TESTE	0	Not Tested	
479	0	2/10/2019	D	33	Alive	7.5	Sorex	minutus	Female	adult	Yes	Yes	Yes	Yes	Yes	704	335	114	58	Am	NEGATIVE	NOT TESTE	0	Not Tested	
478	1	1/10/2019	G	19	Dead	14.2	Apodemus	agrarius	Female	adult	No	Yes	Yes	Yes	Yes	830	660	190	94	Am	POSITIVE	POSITIVE	256	NEGATIVE	
477	1	1/10/2019	G	11	Dead	13.5	Apodemus	agrarius	Male	adult	No	Yes	Yes	Yes	Yes	800	677	190	100	Am	POSITIVE	POSITIVE	512	POSITIVE	
476	0	1/10/2019	F	3	Dead	25.7	Apodemus	flavicolus	Male	adult	No	Yes	Yes	Yes	Yes	886	1006	250	176	Am	NEGATIVE	NOT TESTE	0	Not Tested	
475	1	1/10/2019	G	4	Dead	16.9	Apodemus	agrarius	Male	adult	No	Yes	Yes	Yes	Yes	820	780	210	125	Am	POSITIVE	POSITIVE	1024	POSITIVE	
474	0	1/10/2019	F	2	Dead	10.5	Myodes	glareolus	Male	juvenile	No	Yes	Yes	Yes	Yes	657	274	150	100	Am	NEGATIVE	NOT TESTE	0	Not Tested	
473	1	1/10/2019	E	1	Dead	29.6	Apodemus	flavicolus	Female	adult	No	Yes	Yes	Yes	Yes	897	960	256	164	Am	POSITIVE	POSITIVE	1024	POSITIVE	
472	0	1/10/2019	E	11	Dead	8.7	Sorex	minutus	Female	adult	No	Yes	Yes	Yes	Yes	720	380	126	70	Am	NEGATIVE	NOT TESTE	0	Not Tested	
471	0	1/10/2019	I	10	Dead	34.1	Apodemus	flavicolus	Male	adult	No	Yes	Yes	Yes	Yes	1038	945	253	174	Am	POSITIVE	NEGATIVE	0	Not Testec	Parasites
470	0	1/10/2019	E	20	Dead	17.1	Myodes	glareolus	Male	adult	No	Yes	Yes	Yes	Yes	792	430	167	129	Am	NEGATIVE	NOT TESTE	0	Not Tested	
469	0	1/10/2019	I	11	Dead	15	Myodes	glareolus	Female	adult	No	Yes	Yes	Yes	Yes	782	400	176	147	Am	NEGATIVE	NOT TESTE	0	Not Tested	
468	0	1/10/2019	E	24	Dead	13.8	Myodes	glareolus	Male	adult	No	Yes	Yes	Yes	Yes	792	390	175	130	Am	NEGATIVE	NOT TESTE	0	Not Tested	
467	1	1/10/2019	I	16	Alive	25.3	Apodemus	flavicolus	Female	adult	Yes	Yes	Yes	Yes	Yes	994	1060	253	165	Am	POSITIVE	POSITIVE	1024	NEGATIVE	
466	0	1/10/2019	I	15	Dead	14.5	Myodes	glareolus	Male	adult	No	Yes	Yes	Yes	Yes	772	404	165	134	Am	NEGATIVE	NOT TESTE	0	Not Tested	
465	0	1/10/2019	F	5	Dead	21.4	Myodes	glareolus	Male	adult	No	Yes	Yes	Yes	Yes	900	478	200	142	Am	NEGATIVE	NOT TESTE	0	Not Tested	
464	0	1/10/2019	E	17	Dead	15.3	Myodes	glareolus	Female	adult	No	Yes	Yes	Yes	Yes	854	461	163	127	Am	NEGATIVE	NOT TESTE	0	Not Tested	
463	0	1/10/2019	I	21	Alive	14.6	Myodes	glareolus	Male	adult	Yes	Yes	Yes	Yes	Yes	770	454	170	110	Am	NEGATIVE	NOT TESTE	0	Not Tested	
462	0	1/10/2019	E	12	Alive	14.9	Apodemus	agrarius	Female	adult	Yes	Yes	Yes	Yes	Yes	836	674	206	100	Am	NEGATIVE	NOT TESTE	0	Not Tested	
461	1	1/10/2019	E	9	Alive	22.2	Apodemus	flavicolus	Male	adult	Yes	Yes	Yes	Yes	Yes	944	959	230	167	Am	POSITIVE	POSITIVE	512	POSITIVE	
460	0	1/10/2019	I	5	Alive	14.5	Myodes	glareolus	Female	adult	Yes	Yes	Yes	Yes	Yes	817	370	173	113	Am	NEGATIVE	NOT TESTE	0	Not Tested	
459	0	1/10/2019	I	18	Alive	24.4	Apodemus	flavicolus	Female	adult	Yes	Yes	Yes	Yes	Yes	980	1025	245	180	Am	POSITIVE	NEGATIVE	0	Not Tested	
458	0	1/10/2019	E	14	Alive	30.2	Apodemus	flavicolus	Male	adult	Yes	Yes	Yes	Yes	Yes	1032	1100	257	198	Am	NEGATIVE	NOT TESTE	0	Not Tested	
457	0	1/10/2019	I	17	Alive	24.2	Apodemus	agrarius	Female	adult	Yes	Yes	Yes	Yes	Yes	982	1026	253	167	Am	NEGATIVE	NOT TESTE	0	Not Tested	
456	0	1/10/2019	F	22	Alive	16	Myodes	glareolus	Male	adult	Yes	Yes	Yes	Yes	Yes	880	428	163	123	Am	NEGATIVE	NOT TESTE	0	Not Tested	
455	0	1/10/2019	F	7	Alive	15.6	Myodes	glareolus	Male	adult	Yes	Yes	Yes	Yes	Yes	807	426	176	144	Am	NEGATIVE	NOT TESTE	0	Not Tested	
454	1	1/10/2019	G	11	Alive	12.7	Apodemus	agrarius	Female	adult	Yes	Yes	Yes	Yes	Yes	830	698	178	100	Am	POSITIVE	POSITIVE	64	Not Tested	
453	1	1/10/2019	F	13	Alive	23	Apodemus	flavicolus	Female	juvenile	Yes	Yes	Yes	Yes	Yes	990	920	253	164	Am	POSITIVE	POSITIVE	512	POSITIVE	
452	0	1/10/2019	F	17	Alive	20.3	Apodemus	flavicolus	Female	adult	Yes	Yes	Yes	Yes	Yes	933	967	253	177	Am	NEGATIVE	NOT TESTE	0	Not Tested	
451	0	1/10/2019	F	21	Alive	17	Myodes	glareolus	Male	adult	Yes	Yes	Yes	Yes	Yes	880	457	175	146	Am	NEGATIVE	NOT TESTE	0	Not Tested	
450	0	1/10/2019	G	8	Alive	11.6	Apodemus	agrarius	Male	adult	Yes	Yes	Yes	Yes	Yes	746	627	196	103	Am	NEGATIVE	NOT TESTE	0	Not Tested	
449	0	1/10/2019	E	8	Alive	15.1	Apodemus	agrarius	Female	adult	Yes	Yes	Yes	Yes	Yes	814	666	187	103	Am	NEGATIVE	NOT TESTE	0	Not Testec	Moribund
448	0	1/10/2019	D	42	Alive	14.7	Myodes	glareolus	Male	adult	Yes	Yes	Yes	Yes	Yes	853	441	168	122	Am	NEGATIVE	NOT TESTE	0	Not Tested	
447	1	1/10/2019	D	28	Alive	29.3	Apodemus	flavicolus	Female	adult	Yes	Yes	Yes	Yes	Yes	1073	1065	230	175	Am	POSITIVE	POSITIVE	4096	Not Testec	Tail infection; originally thought to be Myodes and changed to Apodemus. See weight and size.
446	0	1/10/2019	C	35	Alive	16.4	Apodemus	agrarius	Female	adult	Yes	Yes	Yes	Yes	Yes	863	632	159	131	Am	NEGATIVE	NOT TESTE	0	Not Tested	
445	0	1/10/2019	D	43	Dead	8.1	Sorex	minutus	Male	adult	No	Yes	Yes	Yes	Yes	684	394	122	31	Am	NEGATIVE	NOT TESTE	0	Not Tested	
444	1	1/10/2019	C	4	Alive	14.3	Apodemus	agrarius	Female	adult	Yes	Yes	Yes	Yes	Yes	842	589	146	81	Am	POSITIVE	POSITIVE	64	POSITIVE	
443	0	1/10/2019	D	24	Dead	8.4	Sorex	minutus	Male	adult	No	Yes	Yes	Yes	Yes	686	373	111	55	Am	NEGATIVE	NOT TESTE	0	Not Tested	
442	0	1/10/2019	D	50	Dead	15.6	Myodes	glareolus	Female	adult	No	Yes	Yes	Yes	Yes	817	389	164	104	Am	NEGATIVE	NOT TESTE	0	Not Tested	
441	1	1/10/2019	C	34	Alive	16.9	Apodemus	flavicolus	Male	juvenile	Yes	Yes	Yes	Yes	Yes	845	884	200	166	Am	POSITIVE	POSITIVE	128	NEGATIVE	
440	1	1/10/2019	D	7	Dead	16.5	Myodes	glareolus	Female	adult	No	Yes	Yes	Yes	Yes	823	360	170	106	Am	POSITIVE	POSITIVE	128	Not Tested	
439	0	1/10/2019	D	34	Dead	8.5	Sorex	minutus	Male	adult	No	Yes	Yes	Yes	Yes	681	370	129	70	Am	NEGATIVE	NOT TESTE	0	Not Tested	

438	0	1/10/2019	D	9	Alive	13.7	Apodemus	agrarius	Male	adult	Yes	Yes	Yes	Yes	Yes	794	622	157	105	Am	NEGATIVE	NOT TESTE	0	Not Tested	
437	0	1/10/2019	D	19	Dead	9.4	Sorex	minutus	Male	adult	No	Yes	Yes	Yes	Yes	728	373	114	79	Am	NEGATIVE	NOT TESTE	0	Not Tested	
436	0	1/10/2019	C	18	Dead	41.4	Mustela	nivales	Female	adult	No	Yes	Yes	Yes	Yes	1520	395	204	73	Am	NEGATIVE	NOT TESTE	0	Not Testec	Weasel (Mustela nirvalis)
435	0	1/10/2019	C	37	Alive	16	Apodemus	agrarius	Female	adult	Yes	Yes	Yes	Yes	Yes	845	649	189	103	Am	NEGATIVE	NOT TESTE	0	Not Tested	
434	1	1/10/2019	C	43	Dead	13.6	Apodemus	agrarius	Female	adult	No	Yes	Yes	Yes	Yes	726	598	199	197	Am	POSITIVE	POSITIVE	1024	NEGATIVE	
433	0	1/10/2019	D	48	Dead	34.3	Apodemus	flavicolus	Male	adult	No	Yes	Yes	Yes	Yes	884	1053	248	168	Am	NEGATIVE	NEGATIVE	0	Not Testec	Fleas
432	0	1/10/2019	D	39	Alive	16.1	Myodes	glareolus	Male	adult	Yes	Yes	Yes	Yes	Yes	867	503	174	147	Am	POSITIVE	NEGATIVE	0	Not Testec	Moribund
431	0	30/09/2019	F	10	Dead	14.2	Myodes	glareolus	Male	adult	No	Yes	Yes	Yes	Yes	765	395	164	105	Am	NEGATIVE	NOT TESTE	0	Not Tested	
430	0	30/09/2019	F	5	Dead	14.4	Myodes	glareolus	Male	adult	No	Yes	Yes	Yes	Yes	803	418	158	108	Am	NEGATIVE	NOT TESTE	0	Not Tested	
429	0	30/09/2019	F	2	Dead	9.1	Myodes	glareolus	Male	juvenile	No	Yes	Yes	Yes	Yes	706	260	153	80	Am	POSITIVE	NEGATIVE	0	Not Tested	
428	0	30/09/2019	G	25	Dead	15.2	Apodemus	agrarius	Male	adult	No	Yes	Yes	Yes	Yes	865	707	195	108	Am	NEGATIVE	NOT TESTE	0	Not Testec	Tail infection
427	0	30/09/2019	G	4	Dead	13.7	Apodemus	agrarius	Female	adult	No	Yes	Yes	Yes	Yes	762	671	129	112	Am	POSITIVE	NEGATIVE	0	Not Tested	
426	0	30/09/2019	I	9	Dead	16.9	Myodes	glareolus	Male	adult	No	Yes	Yes	Yes	Yes	840	397	143	126	Am	NEGATIVE	NOT TESTE	0	Not Tested	
425	0	30/09/2019	G	19	Dead	31.7	Apodemus	flavicolus	Female	adult	No	Yes	Yes	Yes	Yes	1051	0	238	214	Am	NEGATIVE	NOT TESTE	0	Not Testec	Broken tail
424	0	30/09/2019	G	24	Dead	27.1	Microtus	arvalis	Male	adult	No	Yes	Yes	Yes	Yes	1068	340	150	148	Am	NEGATIVE	NOT TESTE	0	Not Tested	
423	1	30/09/2019	G	10	Dead	12.8	Apodemus	agrarius	Female	adult	No	Yes	Yes	Yes	Yes	764	636	170	93	Am	NEGATIVE	NOT TESTE	128	NEGATIVE	picked up during workshop 1-22 and screened- was not pos in prior two screens
422	0	30/09/2019	G	7	Dead	17.3	Apodemus	agrarius	Male	adult	No	Yes	Yes	Yes	Yes	858	712	175	120	Am	NEGATIVE	NOT TESTE	0	Not Tested	
421	0	30/09/2019	G	8	Dead	15.3	Apodemus	agrarius	Male	adult	No	Yes	Yes	Yes	Yes	843	668	190	102	Am	NEGATIVE	NEGATIVE	0	Not Testec	Mites; picked up in 1-22 workshop and screened by IFA using kidney blood
420	1	30/09/2019	E	20	Dead	15.4	Apodemus	agrarius	Male	adult	No	Yes	Yes	Yes	Yes	828	680	200	106	Am	POSITIVE	POSITIVE	128	NEGATIVE	Tail infection
419	0	30/09/2019	E	11	Dead	15.2	Myodes	glareolus	Male	adult	No	Yes	Yes	Yes	Yes	766	356	152	114	Am	NEGATIVE	NOT TESTE	0	Not Tested	
418	0	30/09/2019	I	15	Dead	12.2	Myodes	glareolus	Male	adult	No	Yes	Yes	Yes	Yes	808	398	138	117	Am	NEGATIVE	NOT TESTE	0	Not Tested	
417	0	30/09/2019	I	3	Dead	15.1	Myodes	glareolus	Male	adult	No	Yes	Yes	Yes	Yes	738	153	155	112	Am	NEGATIVE	NOT TESTE	0	Not Tested	
416	1	30/09/2019	E	17	Dead	16.1	Myodes	glareolus	Male	adult	No	Yes	Yes	Yes	Yes	890	352	152	108	Am	POSITIVE	POSITIVE	256	Not Testec	Mites
415	0	30/09/2019	E	24	Dead	18.7	Myodes	glareolus	Male	adult	No	Yes	Yes	Yes	Yes	907	508	184	125	Am	NEGATIVE	NOT TESTE	0	Not Tested	
414	0	30/09/2019	I	16	Alive	13.6	Myodes	glareolus	Male	adult	Yes	Yes	Yes	Yes	Yes	785	330	163	127	Am	NEGATIVE	NOT TESTE	0	Not Testec	Fleas
413	0	30/09/2019	I	21	Alive	15.4	Myodes	glareolus	Male	adult	Yes	Yes	Yes	Yes	Yes	823	386	153	115	Am	NEGATIVE	NOT TESTE	0	Not Tested	
412	1	30/09/2019	I	20	Alive	21.3	Myodes	glareolus	Male	adult	Yes	Yes	Yes	Yes	Yes	1046	470	154	130	Am	POSITIVE	POSITIVE	128	Not Tested	
411	0	30/09/2019	I	12	Alive	14.9	Myodes	glareolus	Male	juvenile	Yes	Yes	Yes	Yes	Yes	810	389	162	125	Am	NEGATIVE	NOT TESTE	0	Not Tested	
410	0	30/09/2019	I	10	Alive	17.6	Myodes	glareolus	Female	adult	Yes	Yes	Yes	Yes	Yes	906	0	131	147	Am	NEGATIVE	NOT TESTE	0	Not Testec	No tail
409	0	30/09/2019	I	25	Alive	15.2	Myodes	glareolus	Male	adult	Yes	Yes	Yes	Yes	Yes	866	327	162	135	Am	NEGATIVE	NOT TESTE	0	Not Tested	
408	0	30/09/2019	I	7	Alive	17.4	Myodes	glareolus	Female	adult	Yes	Yes	Yes	Yes	Yes	980	373	144	140	Am	NEGATIVE	NOT TESTE	0	Not Testec	Lactating
407	1	30/09/2019	F	8	Alive	29.1	Apodemus	flavicolus	Male	adult	Yes	Yes	Yes	Yes	Yes	1070	998	227	187	Am	POSITIVE	POSITIVE	512	NEGATIVE	
406	0	30/09/2019	F	18	Alive	18.6	Myodes	glareolus	Male	adult	Yes	Yes	Yes	Yes	Yes	869	404	170	133	Am	NEGATIVE	NOT TESTE	0	Not Tested	
405	0	30/09/2019	F	17	Alive	15.7	Myodes	glareolus	Male	adult	Yes	Yes	Yes	Yes	Yes	870	410	174	113	Am	NEGATIVE	NOT TESTE	0	Not Tested	
404	0	30/09/2019	F	13	Alive	29.9	Apodemus	flavicolus	Male	adult	Yes	Yes	Yes	Yes	Yes	1020	1060	247	165	Am	NEGATIVE	NOT TESTE	0	Not Tested	
403	0	30/09/2019	F	6	Alive	13.8	Myodes	glareolus	Male	adult	Yes	Yes	Yes	Yes	Yes	849	419	173	140	Am	NEGATIVE	NOT TESTE	0	Not Tested	
402	0	30/09/2019	F	24	Alive	16.8	Myodes	glareolus	Female	adult	Yes	Yes	Yes	Yes	Yes	909	463	173	126	Am	POSITIVE	NEGATIVE	0	Not Tested	
401	0	30/09/2019	G	6	Alive	11.7	Myodes	glareolus	Male	juvenile	Yes	Yes	Yes	Yes	Yes	770	255	150	115	Am	NEGATIVE	NOT TESTE	0	Not Tested	
400	1	30/09/2019	I	4	Alive	20.2	Myodes	glareolus	Male	adult	Yes	Yes	Yes	Yes	Yes	1006	389	154	120	Am	POSITIVE	POSITIVE	256	Not Tested	
399	1	30/09/2019	G	5	Alive	17.8	Apodemus	agrarius	Female	adult	Yes	Yes	Yes	Yes	Yes	948	729	220	122	Am	POSITIVE	POSITIVE	64	NEGATIVE	
398	1	30/09/2019	E	14	Alive	22.8	Apodemus	flavicolus	Female	adult	Yes	Yes	Yes	Yes	Yes	986	953	266	180	Am	POSITIVE	POSITIVE	32	POSITIVE	retested a couple times during 1-22 workshop- NEG
397	1	30/09/2019	G	1	Alive	15.5	Apodemus	agrarius	Male	adult	Yes	Yes	Yes	Yes	Yes	833	704	177	105	Am	POSITIVE	POSITIVE	8192	POSITIVE	
396	1	30/09/2019	I	22	Alive	20	Apodemus	flavicolus	Male	juvenile	Yes	Yes	Yes	Yes	Yes	968	968	240	180	Am	POSITIVE	POSITIVE	128	POSITIVE	
395	1	30/09/2019	E	9	Alive	13.3	Myodes	glareolus	Male	adult	Yes	Yes	Yes	Yes	Yes	753	458	166	132	Am	NEGATIVE	POSITIVE	1024	Not Tested	
394	1	30/09/2019	E	6	Alive	16.8	Myodes	glareolus	Female	adult	Yes	Yes	Yes	Yes	Yes	880	401	179	159	Am	POSITIVE	POSITIVE	256	Not Tested	
393	0	30/09/2019	F	8	Alive	22.3	Myodes	glareolus	Male	adult	Yes	Yes	Yes	Yes	Yes	1040	515	171	170	Am	NEGATIVE	NOT TESTE	0	Not Tested	
392	0	30/09/2019	D	38	Dead	6.9	Sorex	minutus	Male	adult	No	Yes	Yes	Yes	Yes	685	420	130	55	Am	NEGATIVE	NOT TESTE	0	Not Tested	
391	1	30/09/2019	D	32	Dead	22.5	Apodemus	flavicolus	Male	adult	No	Yes	Yes	Yes	Yes	939	1039	275	175	Am	POSITIVE	POSITIVE	2048	POSITIVE	
390	0	30/09/2019	D	7	Dead	14.1	Myodes	glareolus	Male	adult	No	Yes	Yes	Yes	Yes	893	482	168	131	Am	NEGATIVE	NOT TESTE	0	Not Tested	
389	0	30/09/2019	D	34	Dead	8.1	Sorex	minutus	Male	adult	No	Yes	Yes	Yes	Yes	684	415	140	60	Am	NEGATIVE	NOT TESTE	0	Not Tested	
388	0	30/09/2019	D	42	Dead	8	Sorex	minutus	Male	adult	No	Yes	Yes	Yes	Yes	670	344	103	60	Am	NEGATIVE	NOT TESTE	0	Not Tested	
387	0	30/09/2019	D	19	Dead	7.4	Sorex	minutus	Male	adult	No	Yes	Yes	Yes	Yes	710	388	119	40	Am	NEGATIVE	NOT TESTE	0	Not Tested	
386	0	30/09/2019	D	5	Dead	8.1	Sorex	minutus	Female	adult	No	Yes	Yes	Yes	Yes	630	397	104	40	Am	NEGATIVE	NOT TESTE	0	Not Tested	
385	0	30/09/2019	C	45	Dead	13.9	Myodes	glareolus	Male	adult	No	Yes	Yes	Yes	Yes	774	426	158	148	Am	NEGATIVE	NOT TESTE	0	Not Tested	
384	0	30/09/2019	C	11	Dead	13.2	Myodes	glareolus	Male	juvenile	No	Yes	Yes	Yes	Yes	823	369	163	139	Am	NEGATIVE	NOT TESTE	0	Not Tested	
383	0	30/09/2019	C	43	Alive	17.6	Apodemus	agrarius	Male	adult	Yes	Yes	Yes	Yes	Yes	885	607	162	100	Am	NEGATIVE	NOT TESTE	0	Not Testec	Infected tail
382	1	30/09/2019	C	41	Dead	9.5	Myodes	glareolus	Male	juvenile	No	Yes	Yes	Yes	Yes	648	345	145	115	Am	POSITIVE	POSITIVE	128	Not Tested	
381	0	30/09/2019	C	16	Dead	13.7	Myodes	glareolus	Male	juvenile	No	Yes	Yes	Yes	Yes	398	389	166	165	Am	NEGATIVE	NOT TESTE	0	Not Tested	
380	1	30/09/2019	C	49	Dead	18.7	Myodes	glareolus	Male	adult	No	Yes	Yes	Yes	Yes	957	488	153	169	Am	POSITIVE	POSITIVE	2048	Not Tested	
379	0	30/09/2019	C	29	Dead	7	Sorex	minutus	Female	adult	No	Yes	Yes	Yes	Yes	594	370	87	26	Am	NEGATIVE	NOT TESTE	0	Not Tested	
378	0	30/09/2019	D	8	Alive	15	Apodemus	agrarius	Female	adult	Yes	Yes	Yes	Yes	Yes	819	719	210	119	Am	NEGATIVE	NOT TESTE	0	Not Tested	
377	0	30/09/2019	D	6	Alive	13.3	Myodes	glareolus	Female	adult	Yes	Yes	Yes	Yes	Yes	844	395	171	115	Am	NEGATIVE	NOT TESTE	0	Not Tested	
376	0	30/09/2019	D	25	Alive	20.7	Apodemus	flavicolus	Male	juvenile	Yes	Yes	Yes	Yes	Yes	930	970	220	160	Am	NEGATIVE	NOT TESTE	0	Not Tested	
375	0	30/09/2019	D	43	Alive	26.7	Apodemus	flavicolus	Female	adult	Yes	Yes	Yes	Yes	Yes	1080	1080	264	177	Am	NEGATIVE	NOT TESTE	0	Not Tested	
374	0	30/09/2019	D	22	Alive	21.5	Apodemus	flavicolus	Female	juvenile	Yes	Yes	Yes	Yes	Yes	950	980	244	180	Am	NEGATIVE	NOT TESTE	0	Not Tested	
373	0	30/09/2019	D	24	Alive	13.7	Myodes	glareolus	Male	adult	Yes	Yes	Yes	Yes	Yes	898	440	180	120	Am	NEGATIVE	NOT TESTE	0	Not Tested	
372	0	30/09/2019	D	23	Alive	18.9	Apodemus	agrarius	Male	adult	Yes	Yes	Yes	Yes	Yes	967	940	235	168	Am	NEGATIVE	NOT TESTE	0	Not Tested	
371	0	30/09/2019	D	33	Alive	13.6	Myodes	glareolus	Male	adult	Yes	Yes	Yes	Yes	Yes	890	390	170	130	Am	NEGATIVE	NOT TESTE	0	Not Tested	
370	0	30/09/2019	D	11	Alive	13.6	Apodemus	agrarius	Female	adult	Yes	Yes	Yes	Yes	Yes	830	700	190	110	Am	NEGATIVE	NOT TESTE	0	Not Tested	
369	0	30/09/2019	D	49	Alive	36.8	Apodemus	flavicolus	Male	adult	Yes	Yes	Yes	Yes	Yes	1155	1120	280	188	Am	NEGATIVE	NOT TESTE	0	Not Tested	
368	0	30/09/2019	D	50	Alive	16.6	Myodes	glareolus	Male	adult	Yes	Yes	Yes	Yes	Yes	855	427	186	140	Am	NEGATIVE	NOT TESTE	0	Not Tested	
367	0	30/09/2019	C	30	Alive	13.9	Myodes	glareolus	Male	adult	Yes	Yes	Yes	Yes	Yes	871	433	167	133	Am	NEGATIVE	NEGATIVE	0	Not Tested	
366	0	30/09/2019	C	34	Alive	12.7	Myodes	glareolus	Female	adult	Yes	Yes	Yes	Yes	Yes	822	410	170	170	Am	NEGATIVE	NOT TESTE	0	Not Testec	Moribund
365	0	30/09/2019	C	42	Alive	14.4	Myodes	glareolus	Male	adult	Yes	Yes	Yes	Yes	Yes	891	420	170	133	Am	NEGATIVE	NOT TESTE	0	Not Tested	
364	0	30/09/2019	C	48	Alive	17.8	Apodemus	flavicolus	Male	juvenile	Yes	Yes	Yes	Yes	Yes	888	920	240	160	Am	POSITIVE	NEGATIVE	0	Not Tested	
363	0	30/09/2019	C	18	Alive	14.6	Apodemus	agrarius	Female	adult	Yes	Yes	Yes	Yes	Yes	870	710	206	110	Am	NEGATIVE	NOT TESTE	0	Not Tested	
362	0	30/09/2019	C	47	Alive	22.4	Apodemus	flavicolus	Male	adult	Yes	Yes	Yes	Yes	Yes	985	885	244	165	Am	NEGATIVE	NOT TESTE	0	Not Tested	
361	1	30/09/2019	C	25	Alive	10	Myodes	glareolus	Male	juvenile	Yes	Yes	Yes	Yes	Yes	779	375	166	110	Am	NEGATIVE	NEGATIVE	0	POSITIVE	Moribund
360	0	30/09/2019	C	37	Alive	12.6	Myodes	glareolus	Female	adult	Yes	Yes	Yes	Yes	Yes	776	409	176	120	Am	NEGATIVE	NOT TESTE	0	Not Testec	Moribund
359	0	30/09/2019	D	1	Alive	15	Myodes	glareolus	Male	adult	Yes	Yes	Yes	Yes	Yes	899	395	180	124	Am	NEGATIVE	NOT TESTE	0	Not Tested	
358	0	30/09/2019	D	28	Alive	13	Apodemus	agrarius	Female	adult	Yes	Yes	Yes	Yes	Yes	844	600	199	129	Am	NEGATIVE	NOT TESTE	0	Not Tested	
357	0	29/09/2019	G	2	Dead	13.9	Apodemus	agrarius	Female	adult	Yes	Yes	Yes	Yes	Yes	850	597	146	105	Pm	NEGATIVE	NOT TESTE	0	Not Testec	Moribund, Little blood drawn
356	1	29/09/2019	H	17	Alive	13.7	Apodemus	agrarius	Male	adult	Yes	Yes	Yes	Yes	Yes	655	671	172	78	Pm	POSITIVE	POSITIVE	256	NEGATIVE	
355	1	29/09/2019	F	13	Alive	23.7	Myodes	glareolus	Male	adult	Yes	Yes	Yes	Yes	Yes	925	475	150	110	Pm	POSITIVE	POSITIVE	2048	Not Tested	
354	0	29/09/2019	F	23	Dead	15.4	Myodes	glareolus	Male	adult	No	Yes	Yes	Yes	Yes	810	390	166	106	Am	NEGATIVE	NOT TESTE	0	Not Testec	Tail infection
353	0	29/09/2019	F	6	Dead	15.6	Myodes	glareolus	Female	adult	No	Yes	Yes	Yes	Yes	794	425	158	114	Am	NEGATIVE	NOT TESTE	0	Not Tested	
352	0	29/09/2019	E	9	Dead	16.2	Myodes	glareolus	Female	adult	No	Yes	Yes	Yes	Yes	810	369	156	134	Am	NEGATIVE	NOT TESTE	0	Not Tested	
351	1	29/09/2019	G	4	Dead	17.7	Apodemus	agrarius	Female	adult	No	Yes	Yes	Yes	Yes	907	713	186	113	Am	POSITIVE	POSITIVE	256	POSITIVE	
350	0	29/09/2019	F	17	Dead	14.8	Myodes	glareolus	Male	adult	No	Yes	Yes	Yes	Yes	779	462	174	138	Am	NEGATIVE	NOT TESTE	0	Not Tested	

349	0	29/09/2019	E	21	Dead	15.2	Myodes	glareolus	Female	adult	No	Yes	Yes	Yes	Yes	763	425	163	113	Am	NEGATIVE	NOT TESTE	0	Not Tested	
348	0	29/09/2019	F	5	Dead	15.1	Myodes	glareolus	Female	adult	No	Yes	Yes	Yes	Yes	744	363	180	175	Am	NEGATIVE	NOT TESTE	0	Not Tested	
347	0	29/09/2019	E	19	Dead	15.1	Myodes	glareolus	Male	adult	No	Yes	Yes	Yes	Yes	726	440	142	98	Am	NEGATIVE	NOT TESTE	0	Not Testec	Fleas
346	1	29/09/2019	F	15	Dead	16	Myodes	glareolus	Male	adult	No	Yes	Yes	Yes	Yes	746	424	146	110	Am	POSITIVE	POSITIVE	64	Not Tested	
345	1	29/09/2019	G	25	Dead	15.6	Apodemus	agrarius	Male	adult	No	Yes	Yes	Yes	Yes	860	636	165	90	Am	POSITIVE	POSITIVE	2048	NEGATIVE	
344	0	29/09/2019	E	12	Dead	15.3	Myodes	glareolus	Male	adult	No	Yes	Yes	Yes	Yes	821	363	164	105	Am	NEGATIVE	NOT TESTE	0	Not Tested	
343	1	29/09/2019	G	19	Dead	5.3	Apodemus	agrarius	Male	juvenile	No	Yes	Yes	Yes	Yes	520	418	146	172	Am	POSITIVE	POSITIVE	4096	POSITIVE	
342	0	29/09/2019	G	15	Dead	17.8	Myodes	glareolus	Female	adult	No	Yes	Yes	Yes	Yes	893	302	156	111	Am	NEGATIVE	NOT TESTE	0	Not Tested	
341	0	29/09/2019	G	1	Dead	14.8	Apodemus	agrarius	Male	adult	No	Yes	Yes	Yes	Yes	790	682	155	77	Am	NEGATIVE	NOT TESTE	0	Not Testec	Moribund, no liquid in Lungs tube
340	0	29/09/2019	G	3	Alive	15.4	Apodemus	agrarius	Male	adult	Yes	Yes	Yes	Yes	Yes	885	585	188	90	Am	NEGATIVE	NOT TESTE	0	Not Tested	
339	0	29/09/2019	E	8	Alive	14.8	Myodes	glareolus	Female	adult	Yes	Yes	Yes	Yes	Yes	855	316	137	113	Am	NEGATIVE	NOT TESTE	0	Not Tested	
338	1	29/09/2019	E	14	Alive	15.7	Myodes	glareolus	Female	adult	Yes	Yes	Yes	Yes	Yes	930	417	174	89	Am	POSITIVE	POSITIVE	512	Not Tested	
337	0	29/09/2019	E	7	Alive	13.8	Apodemus	flavicolus	Male	juvenile	Yes	Yes	Yes	Yes	Yes	769	817	225	149	Am	NEGATIVE	NOT TESTE	0	Not Tested	
336	0	29/09/2019	E	18	Alive	19.4	Myodes	glareolus	Male	adult	Yes	Yes	Yes	Yes	Yes	954	465	154	138	Am	NEGATIVE	NOT TESTE	0	Not Tested	
335	0	29/09/2019	F	1	Alive	20.2	Apodemus	flavicolus	Male	juvenile	Yes	Yes	Yes	Yes	Yes	908	910	237	165	Am	NEGATIVE	NOT TESTE	0	Not Tested	
334	1	29/09/2019	E	15	Alive	19.5	Myodes	glareolus	Female	adult	Yes	Yes	Yes	Yes	Yes	979	478	169	154	Am	POSITIVE	POSITIVE	8192	Not Testec	Breastfeeding (lactating), little blood taken, Moribund
333	0	29/09/2019	E	13	Alive	15.9	Myodes	glareolus	Male	adult	Yes	Yes	Yes	Yes	Yes	874	356	156	124	Am	NEGATIVE	NOT TESTE	0	Not Tested	
332	0	29/09/2019	F	21	Alive	24	Apodemus	flavicolus	Male	adult	Yes	Yes	Yes	Yes	Yes	998	960	245	184	Am	NEGATIVE	NEGATIVE	0	Not Tested	
331	0	29/09/2019	F	8	Alive	31.2	Apodemus	flavicolus	Male	adult	Yes	Yes	Yes	Yes	Yes	1058	1001	210	185	Am	NEGATIVE	NOT TESTE	0	Not Tested	
330	0	29/09/2019	F	22	Alive	24.6	Apodemus	flavicolus	Male	adult	Yes	Yes	Yes	Yes	Yes	995	948	236	164	Am	NEGATIVE	NEGATIVE	0	Not Tested	
329	1	29/09/2019	F	25	Alive	28.8	Apodemus	flavicolus	Male	adult	Yes	Yes	Yes	Yes	Yes	1011	951	237	172	Am	POSITIVE	POSITIVE	1024	NEGATIVE	Mites
328	0	29/09/2019	F	10	Alive	30.9	Apodemus	flavicolus	Male	adult	Yes	Yes	Yes	Yes	Yes	1075	1055	256	155	Am	NEGATIVE	NOT TESTE	0	Not Tested	
327	0	29/09/2019	F	7	Alive	26.1	Apodemus	flavicolus	Male	adult	Yes	Yes	Yes	Yes	Yes	1003	0	244	167	Am	NEGATIVE	NOT TESTE	0	Not Testec	Missing part of tail
326	1	29/09/2019	G	8	Alive	17.6	Apodemus	agrarius	Male	adult	Yes	Yes	Yes	Yes	Yes	898	675	202	109	Am	POSITIVE	POSITIVE	64	POSITIVE	
325	0	29/09/2019	E	24	Alive	33.5	Apodemus	flavicolus	Male	adult	Yes	Yes	Yes	Yes	Yes	1059	0	256	150	Am	NEGATIVE	NOT TESTE	0	Not Testec	Broken tail
324	0	29/09/2019	E	24	Alive	35.5	Apodemus	flavicolus	Female	adult	Yes	Yes	Yes	Yes	Yes	1105	1002	252	192	Am	NEGATIVE	NOT TESTE	0	Not Testec	Helminth eggs
323	0	29/09/2019	E	11	Alive	15.1	Myodes	glareolus	Female	adult	Yes	Yes	Yes	Yes	Yes	773	399	148	125	Am	NEGATIVE	NOT TESTE	0	Not Tested	
322	0	29/09/2019	F	24	Dead	15.9	Myodes	glareolus	Male	adult	No	Yes	Yes	Yes	Yes	807	416	180	116	Am	NEGATIVE	NOT TESTE	0	Not Testec	Moribund
321	1	29/09/2019	F	24	Alive	13.6	Myodes	glareolus	Female	adult	Yes	Yes	Yes	Yes	Yes	841	413	171	124	Am	POSITIVE	POSITIVE	8192	Not Tested	
320	1	29/09/2019	C	42	Dead	8.3	Myodes	glareolus	Male	juvenile	No	Yes	Yes	Yes	Yes	599	226	164	113	Am	POSITIVE	POSITIVE	128	Not Tested	
319	0	29/09/2019	C	18	Dead	13.3	Myodes	glareolus	Female	adult	No	Yes	Yes	Yes	Yes	720	379	153	123	Am	NEGATIVE	NOT TESTE	0	Not Tested	
318	1	29/09/2019	C	47	Dead	12.7	Myodes	glareolus	Male	adult	No	Yes	Yes	Yes	Yes	725	363	164	105	Am	POSITIVE	POSITIVE	256	NEGATIVE	used kidney for IFA titer
317	0	29/09/2019	C	11	Dead	16	Myodes	glareolus	Female	adult	No	Yes	Yes	Yes	Yes	811	426	168	119	Am	NEGATIVE	NOT TESTE	0	Not Tested	
316	0	29/09/2019	C	30	Dead	8.5	Sorex	minutus	Male	adult	No	Yes	Yes	Yes	Yes	696	377	125	71	Am	NEGATIVE	NOT TESTE	0	Not Tested	
315	1	29/09/2019	C	45	Dead	8.8	Myodes	glareolus	Male	juvenile	No	Yes	Yes	Yes	Yes	637	327	163	104	Am	POSITIVE	POSITIVE	128	Not Tested	
314	0	29/09/2019	C	43	Dead	7.8	Sorex	minutus	Female	adult	No	Yes	Yes	Yes	Yes	672	412	138	60	Am	NEGATIVE	NOT TESTE	0	Not Tested	
313	0	29/09/2019	C	27	Dead	7.8	Sorex	minutus	Male	adult	No	Yes	Yes	Yes	Yes	655	342	113	65	Am	NEGATIVE	NOT TESTE	0	Not Tested	
311	0	29/09/2019	C	34	Dead	7.3	Sorex	minutus	Male	adult	No	Yes	Yes	Yes	Yes	678	363	134	56	Am	NEGATIVE	NOT TESTE	0	Not Tested	
310	0	29/09/2019	C	41	Dead	8.7	Myodes	glareolus	Female	juvenile	No	Yes	Yes	Yes	Yes	603	305	165	94	Am	POSITIVE	NEGATIVE	0	Not Tested	
309	0	29/09/2019	D	17	Dead	6.02	Sorex	minutus	Male	adult	No	Yes	Yes	Yes	Yes	650	322	155	64	Am	NEGATIVE	NOT TESTE	0	Not Tested	
308	1	29/09/2019	D	37	Dead	18.2	Myodes	glareolus	Male	adult	No	Yes	Yes	Yes	Yes	902	453	164	109	Am	POSITIVE	POSITIVE	128	Not Tested	
307	0	29/09/2019	D	29	Dead	7.4	Sorex	minutus	Male	adult	No	Yes	Yes	Yes	Yes	679	327	124	48	Am	NEGATIVE	NOT TESTE	0	Not Tested	
306	0	29/09/2019	D	2	Dead	13	Apodemus	agrarius	Male	adult	No	Yes	Yes	Yes	Yes	715	655	187	112	Am	NEGATIVE	NEGATIVE	0	Not Tested	
305	0	29/09/2019	D	43	Dead	4.8	Myodes	glareolus	Female	adult	No	Yes	Yes	Yes	Yes	759	363	151	109	Am	NEGATIVE	NOT TESTE	0	Not Tested	
304	0	29/09/2019	D	45	Dead	7.5	Sorex	minutus	Male	adult	No	Yes	Yes	Yes	Yes	589	141	113	166	Am	NEGATIVE	NOT TESTE	0	Not Tested	
303	0	29/09/2019	C	44	Alive	16.6	Myodes	glareolus	Male	adult	Yes	Yes	Yes	Yes	Yes	879	362	176	155	Am	NEGATIVE	NOT TESTE	0	Not Tested	
302	0	29/09/2019	C	29	Alive	23.5	Myodes	glareolus	Female	adult	Yes	Yes	Yes	Yes	Yes	1006	431	121	131	Am	NEGATIVE	NOT TESTE	0	Not Tested	
301	0	29/09/2019	C	37	Alive	11.9	Myodes	glareolus	Female	adult	Yes	Yes	Yes	Yes	Yes	850	316	169	105	Am	NEGATIVE	NOT TESTE	0	Not Tested	
300	0	29/09/2019	C	25	Alive	17.8	Apodemus	flavicolus	Male	juvenile	Yes	Yes	Yes	Yes	Yes	885	846	210	165	Am	NEGATIVE	NOT TESTE	0	Not Tested	
299	0	29/09/2019	C	19	Alive	12.3	Myodes	glareolus	Male	juvenile	Yes	Yes	Yes	Yes	Yes	827	392	166	106	Am	NEGATIVE	NOT TESTE	0	Not Tested	
298	0	29/09/2019	C	8	Alive	26.3	Apodemus	flavicolus	Male	adult	Yes	Yes	Yes	Yes	Yes	973	1002	235	166	Am	NEGATIVE	NOT TESTE	0	Not Tested	
297	0	29/09/2019	C	48	Alive	16.5	Apodemus	flavicolus	Female	juvenile	Yes	Yes	Yes	Yes	Yes	850	896	222	165	Am	NEGATIVE	NOT TESTE	0	Not Tested	
296	0	29/09/2019	D	1	Alive	22.2	Apodemus	flavicolus	Male	juvenile	Yes	Yes	Yes	Yes	Yes	954	1002	229	167	Am	NEGATIVE	NOT TESTE	0	Not Tested	
295	0	29/09/2019	D	6	Alive	14.2	Apodemus	agrarius	Female	juvenile	Yes	Yes	Yes	Yes	Yes	854	642	175	101	Am	NEGATIVE	NOT TESTE	0	Not Tested	
294	0	29/09/2019	D	42	Alive	34.9	Apodemus	flavicolus	Male	adult	Yes	Yes	Yes	Yes	Yes	1074	1071	250	170	Am	NEGATIVE	NOT TESTE	0	Not Testec	Fly eggs
293	0	29/09/2019	D	23	Alive	19.8	Apodemus	flavicolus	Female	juvenile	Yes	Yes	Yes	Yes	Yes	904	952	227	135	Am	NEGATIVE	NOT TESTE	0	Not Tested	
292	0	29/09/2019	D	19	Alive	33.3	Apodemus	flavicolus	Male	adult	Yes	Yes	Yes	Yes	Yes	1086	996	262	174	Am	NEGATIVE	NOT TESTE	0	Not Tested	
291	0	29/09/2019	D	32	Alive	14.7	Apodemus	agrarius	Male	adult	Yes	Yes	Yes	Yes	Yes	806	579	179	116	Am	NEGATIVE	NOT TESTE	0	Not Testec	Blood and muscle tubes are mixed
290	0	29/09/2019	D	11	Alive	23.5	Apodemus	flavicolus	Male	adult	Yes	Yes	Yes	Yes	Yes	948	884	243	169	Am	POSITIVE	NEGATIVE	0	Not Tested	
289	0	29/09/2019	D	7	Alive	14.1	Apodemus	agrarius	Male	adult	Yes	Yes	Yes	Yes	Yes	846	642	182	107	Am	NEGATIVE	NOT TESTE	0	NEGATIVE	
288	1	29/09/2019	D	14	Alive	34.8	Apodemus	flavicolus	Male	adult	Yes	Yes	Yes	Yes	Yes	1079	1018	250	198	Am	POSITIVE	POSITIVE	1024	POSITIVE	
287	0	29/09/2019	D	28	Alive	28.5	Apodemus	flavicolus	Male	adult	Yes	Yes	Yes	Yes	Yes	955	976	236	166	Am	NEGATIVE	NOT TESTE	0	Not Tested	
286	1	29/09/2019	D	33	Alive	14.1	Apodemus	agrarius	Male	adult	Yes	Yes	Yes	Yes	Yes	848	580	185	119	Am	POSITIVE	POSITIVE	128	Not Tested	
285	0	29/09/2019	D	34	Alive	13.6	Myodes	glareolus	Female	adult	Yes	Yes	Yes	Yes	Yes	843	338	156	121	Am	NEGATIVE	NOT TESTE	0	Not Tested	
284	1	29/09/2019	D	38	Alive	32.2	Apodemus	flavicolus	Male	adult	Yes	Yes	Yes	Yes	Yes	1049	988	241	184	Am	POSITIVE	POSITIVE	64	Not Tested	
283	0	29/09/2019	D	25	Alive	7.4	Sorex	minutus	Female	adult	Yes	Yes	Yes	Yes	Yes	600	362	124	68	Am	NEGATIVE	NOT TESTE	0	Not Tested	
282	0	26/09/2019	A	13	Alive	23.8	Apodemus	flavicolus	Female	adult	Yes	Yes	Yes	Yes	Yes	992	908	245	168	Pm	NEGATIVE	NOT TESTE	0	Not Tested	
281	0	26/09/2019	A	49	Dead	13.6	Myodes	glareolus	Male	adult	No	Yes	Yes	Yes	Yes	728	443	160	123	Pm	NEGATIVE	NOT TESTE	0	Not Tested	
280	0	26/09/2019	A	40	Alive	22.4	Apodemus	flavicolus	Male	adult	Yes	Yes	Yes	Yes	Yes	953	875	238	167	Pm	NEGATIVE	NOT TESTE	0	Not Tested	
279	0	26/09/2019	A	44	Alive	26.6	Apodemus	flavicolus	Male	adult	Yes	Yes	Yes	Yes	Yes	999	968	249	162	Pm	NEGATIVE	NOT TESTE	0	Not Tested	
278	0	26/09/2019	A	46	Alive	13.5	Apodemus	agrarius	Male	adult	Yes	Yes	Yes	Yes	Yes	835	648	197	116	Pm	NEGATIVE	NOT TESTE	0	Not Tested	
277	1	26/09/2019	A	16	Alive	21.7	Apodemus	flavicolus	Female	adult	Yes	Yes	Yes	Yes	Yes	942	899	241	171	Pm	POSITIVE	POSITIVE	64	NEGATIVE	
276	0	26/09/2019	A	14	Alive	30.9	Apodemus	flavicolus	Male	adult	Yes	Yes	Yes	Yes	Yes	1053	1106	261	188	Pm	NEGATIVE	NOT TESTE	0	Not Tested	
275	0	26/09/2019	A	12	Alive	14.6	Apodemus	agrarius	Female	adult	Yes	Yes	Yes	Yes	Yes	728	640	168	101	Pm	NEGATIVE	NOT TESTE	0	Not Tested	
274	1	26/09/2019	A	9	Dead	15.5	Myodes	glareolus	Male	adult	No	Yes	Yes	Yes	Yes	854	414	172	114	Pm	POSITIVE	POSITIVE	256	Not Tested	
273	0	26/09/2019	A	36	Dead	14.8	Myodes	glareolus	Male	adult	No	Yes	Yes	Yes	Yes	782	425	171	116	Pm	NEGATIVE	NOT TESTE	0	Not Tested	
272	1	26/09/2019	A	35	Alive	7.3	Mus	musculus	Male	juvenile	Yes	Yes	Yes	Yes	Yes	661	575	143	107	Pm	POSITIVE	POSITIVE	512	Not Tested	
271	0	26/09/2019	A	24	Dead	6	Sorex	minutus	Female	adult	No	Yes	Yes	Yes	Yes	705	357	123	81	Pm	NEGATIVE	NOT TESTE	0	Not Tested	
270	1	26/09/2019	A	22	Alive	30.5	Apodemus	flavicolus	Male	adult	Yes	Yes	Yes	Yes	Yes	1043	1151	240	187	Pm	POSITIVE	POSITIVE	512	NEGATIVE	
269	0	26/09/2019	A	2	Dead	7.7	Sorex	minutus	Male	adult	No	Yes	Yes	Yes	Yes	701	407	124	58	Pm	NEGATIVE	NOT TESTE	0	Not Tested	
268	0	26/09/2019	A	38	Alive	27.8	Apodemus	flavicolus	Female	adult	Yes	Yes	Yes	Yes	Yes	1059	1026	236	166	Pm	NEGATIVE	NOT TESTE	0	Not Testec	Fleas
267	0	26/09/2019	A	3	Dead	8	Sorex	minutus	Male	adult	No	Yes	Yes	Yes	Yes	617	391	136	55	Am	NEGATIVE	NOT TESTE	0	Not Testec	2 shrews in one trap; one half eaten and was not given a number
266	0	26/09/2019	A	50	Dead	14.5	Myodes	glareolus	Male	juvenile	No	Yes	Yes	Yes	Yes	776	417	176	117	Am	NEGATIVE	NOT TESTE	0	Not Tested	
265	0	26/09/2019	C	33	Dead	23.8	Apodemus	flavicolus	Female	adult	No	Yes	Yes	Yes	Yes	854	1005	238	173	Am	NEGATIVE	NOT TESTE	0	Not Tested	
264	0	26/09/2019	C	34	Dead	14.2	Myodes	glareolus	Male	adult	No	Yes	Yes	Yes	Yes	777	364	158	125	Am	NEGATIVE	NOT TESTE	0	Not Tested	
263	0	26/09/2019	A	25	Alive	34.2	Apodemus	flavicolus	Female	adult	Yes	Yes	Yes	Yes	Yes	1079	1094	231	186	Am	NEGATIVE	NOT TESTE	0	Not Tested	
262	0	26/09/2019	C	31	Dead	14.5	Myodes	glareolus	Male	adult	No	Yes	Yes	Yes	Yes	880	412	174	126	Am	NEGATIVE	NEGATIVE	0	Not Tested	
261	0	26/09/2019	A	5	Alive	20.9	Apodemus	flavicolus	Male	adult	Yes	Yes	Yes	Yes	Yes	795	834	236	166	Am	NEGATIVE	NOT TESTE	0	Not Testec	Marked as 262
260	1	26/09/2019	A	4	Alive	18.2	Apodemus	agrarius	Female	adult	Yes	Yes	Yes	Yes	Yes	955	780	191	106	Am	POSITIVE	POSITIVE	128	POSITIVE	

259	0	26/09/2019	C	35	Alive	13.4	Myodes	glareolus	Male	adult	No	Yes	Yes	Yes	Yes	778	367	165	123	Am	NEGATIVE	NOT TESTE	0	Not Tested	Flea talpa
258	1	26/09/2019	C	30	Dead	21.7	Myodes	glareolus	Male	adult	No	Yes	Yes	Yes	Yes	973	482	164	147	Am	POSITIVE	POSITIVE	1024	Not Tested	
257	1	26/09/2019	A	17	Alive	24.4	Apodemus	agrarius	Male	adult	Yes	Yes	Yes	Yes	Yes	950	707	186	109	Am	POSITIVE	POSITIVE	4096	POSITIVE	
256	1	26/09/2019	C	39	Dead	14.3	Myodes	glareolus	Female	adult	No	Yes	Yes	Yes	Yes	837	407	171	139	Am	POSITIVE	POSITIVE	1024	Not Tested	Marked as 257
255	0	26/09/2019	A	20	Alive	13.8	Apodemus	flavicolus	Female	juvenile	Yes	Yes	Yes	Yes	Yes	783	784	214	146	Am	NEGATIVE	NOT TESTE	0	Not Tested	Mites
254	0	26/09/2019	A	11	Alive	25.5	Apodemus	flavicolus	Female	adult	Yes	Yes	Yes	Yes	Yes	990	927	246	174	Am	NEGATIVE	NOT TESTE	0	Not Tested	
253	0	26/09/2019	B	2	Dead	31.2	Apodemus	flavicolus	Female	adult	No	Yes	Yes	Yes	Yes	929	1039	249	184	Am	NEGATIVE	NOT TESTE	0	Not Tested	
252	1	26/09/2019	C	42	Alive	9.1	Myodes	glareolus	Male	juvenile	Yes	Yes	Yes	Yes	Yes	657	334	157	83	Am	POSITIVE	POSITIVE	256	Not Tested	obtained IFA titer using kidney
251	0	26/09/2019	A	30	Alive	14.8	Myodes	glareolus	Male	adult	Yes	Yes	Yes	Yes	Yes	796	424	156	123	Am	NEGATIVE	NOT TESTE	0	Not Tested	
250	0	26/09/2019	B	10	Dead	14.3	Myodes	glareolus	Male	juvenile	No	Yes	Yes	Yes	Yes	759	397	176	124	Am	NEGATIVE	NOT TESTE	0	Not Tested	
249	0	26/09/2019	A	44	Alive	10.9	Muscardin	aranus	Female	adult	Yes	Yes	Yes	Yes	Yes	749	625	158	91	Am	POSITIVE	NEGATIVE	0	Not Tested	Dormouse, little blood drawn
247	0	26/09/2019	B	18	Alive	16.1	Apodemus	flavicolus	Male	juvenile	Yes	Yes	Yes	Yes	Yes	778	855	215	168	Am	NEGATIVE	NOT TESTE	0	Not Tested	
246	0	26/09/2019	B	36	Alive	15.8	Myodes	glareolus	Male	adult	Yes	Yes	Yes	Yes	Yes	836	411	169	132	Am	NEGATIVE	NOT TESTE	0	Not Tested	
245	0	26/09/2019	B	28	Dead	25.6	Apodemus	flavicolus	Male	adult	No	Yes	Yes	Yes	Yes	904	730	234	176	Am	NEGATIVE	NOT TESTE	0	Not Tested	Fleas
244	0	26/09/2019	B	42	Alive	17.2	Myodes	glareolus	Male	juvenile	Yes	Yes	Yes	Yes	Yes	833	406	168	125	Am	NEGATIVE	NOT TESTE	0	Not Tested	Little blood
243	1	26/09/2019	C	36	Dead	20.3	Myodes	glareolus	Male	adult	No	Yes	Yes	Yes	Yes	903	482	164	130	Am	POSITIVE	POSITIVE	4096	Not Tested	
242	0	26/09/2019	B	46	Alive	17.3	Myodes	glareolus	Male	adult	Yes	Yes	Yes	Yes	Yes	880	394	156	122	Am	NEGATIVE	NOT TESTE	0	Not Tested	
241	0	26/09/2019	B	26	Alive	15.6	Myodes	glareolus	Male	adult	Yes	Yes	Yes	Yes	Yes	790	407	173	139	Am	NEGATIVE	NOT TESTE	0	Not Tested	Fleas
240	0	26/09/2019	B	32	Alive	23.3	Apodemus	flavicolus	Male	adult	Yes	Yes	Yes	Yes	Yes	925	879	243	167	Am	NEGATIVE	NOT TESTE	0	Not Tested	
239	1	26/09/2019	C	11	Dead	23	Myodes	glareolus	Male	adult	No	Yes	Yes	Yes	Yes	925	517	169	135	Am	POSITIVE	POSITIVE	512	Not Tested	
238	0	26/09/2019	B	31	Alive	22.1	Myodes	glareolus	Female	adult	Yes	Yes	Yes	Yes	Yes	872	535	167	138	Am	NEGATIVE	NOT TESTE	0	Not Tested	Little blood drawn
237	0	26/09/2019	B	19	Alive	21	Apodemus	flavicolus	Female	adult	Yes	Yes	Yes	Yes	Yes	904	910	230	166	Am	NEGATIVE	NOT TESTE	0	Not Tested	
236	0	26/09/2019	B	1	Alive	30	Apodemus	flavicolus	Male	adult	Yes	Yes	Yes	Yes	Yes	978	992	235	186	Am	POSITIVE	NEGATIVE	0	Not Tested	Mites
235	0	26/09/2019	B	50	Alive	24	Apodemus	flavicolus	Female	adult	Yes	Yes	Yes	Yes	Yes	885	950	254	166	Am	NEGATIVE	NOT TESTE	0		
234	0	26/09/2019	B	41	Alive	21	Apodemus	flavicolus	Female	adult	Yes	Yes	Yes	Yes	Yes	876	989	240	182	Am	POSITIVE	NEGATIVE	0	Not Tested	Fleas, talpa
233	1	26/09/2019	C	5	Alive	33.6	Apodemus	flavicolus	Male	adult	Yes	Yes	Yes	Yes	Yes	1068	1036	242	195	Am	POSITIVE	POSITIVE	512	NEGATIVE	
232	0	26/09/2019	C	9	Alive	9.3	Mus	musculus	Male	adult	Yes	Yes	Yes	Yes	Yes	680	649	157	126	Am	NEGATIVE	NOT TESTE	0	Not Tested	Mus musculus
231	1	26/09/2019	C	7	Alive	14.1	Apodemus	agrarius	Male	adult	Yes	Yes	Yes	Yes	Yes	799	622	162	119	Am	POSITIVE	POSITIVE	64	POSITIVE	
230	0	26/09/2019	C	37	Alive	16.4	Apodemus	agrarius	Male	adult	Yes	Yes	Yes	Yes	Yes	782	681	197	93	Am	NEGATIVE	NOT TESTE	0	Not Tested	
229	0	26/09/2019	C	24	Alive	30	Apodemus	flavicolus	Male	adult	Yes	Yes	Yes	Yes	Yes	981	1005	232	168	Am	NEGATIVE	NOT TESTE	0	Not Tested	
228	0	26/09/2019	C	6	Alive	21.5	Apodemus	flavicolus	Female	adult	Yes	Yes	Yes	Yes	Yes	869	897	238	173	Am	POSITIVE	NEGATIVE	0	Not Tested	
227	0	26/09/2019	D	34	Dead	14.9	Myodes	glareolus	Male	adult	No	Yes	Yes	Yes	Yes	796	375	172	110	Am	NEGATIVE	NOT TESTE	0	Not Tested	
226	0	26/09/2019	C	10	Alive	20.5	Apodemus	flavicolus	Male	adult	Yes	Yes	Yes	Yes	Yes	957	161	222	161	Am	NEGATIVE	NOT TESTE	0	Not Tested	
225	0	26/09/2019	C	27	Alive	15.2	Myodes	glareolus	Female	adult	Yes	Yes	Yes	Yes	Yes	813	388	169	140	Am	NEGATIVE	NOT TESTE	0	Not Tested	
224	0	26/09/2019	D	38	Alive	30.1	Apodemus	flavicolus	Male	adult	Yes	Yes	Yes	Yes	Yes	947	981	246	182	Am	POSITIVE	NEGATIVE	128	NEGATIVE	
223	0	26/09/2019	D	33	Dead	30	Apodemus	flavicolus	Female	adult	No	Yes	Yes	Yes	Yes	895	1120	235	184	Am	NEGATIVE	NOT TESTE	0	Not Tested	Mites
222	0	26/09/2019	D	29	Alive	13.7	Myodes	glareolus	Female	adult	Yes	Yes	Yes	Yes	Yes	726	413	153	123	Am	NEGATIVE	NOT TESTE	0	Not Tested	Little blood drawn
221	0	26/09/2019	D	37	Alive	16.2	Apodemus	flavicolus	Male	adult	Yes	Yes	Yes	Yes	Yes	839	396	171	115	Am	NEGATIVE	NOT TESTE	0	Not Tested	
220	1	26/09/2019	D	21	Alive	32	Apodemus	flavicolus	Male	adult	Yes	Yes	Yes	Yes	Yes	930	1104	231	180	Am	POSITIVE	POSITIVE	64	POSITIVE	Mites, fleas
219	0	26/09/2019	D	46	Alive	11.7	Apodemus	agrarius	Female	juvenile	Yes	Yes	Yes	Yes	Yes	736	575	167	92	Am	NEGATIVE	NOT TESTE	0	Not Tested	Flea
218	0	26/09/2019	D	39	Alive	18.1	Myodes	glareolus	Male	adult	Yes	Yes	Yes	Yes	Yes	870	461	185	133	Am	NEGATIVE	NOT TESTE	0	Not Tested	Moribund
217	0	26/09/2019	D	47	Alive	24.9	Apodemus	flavicolus	Female	adult	Yes	Yes	Yes	Yes	Yes	908	960	246	184	Am	NEGATIVE	NOT TESTE	0	Not Tested	Fleas, mites
216	0	26/09/2019	D	28	Alive	40.5	Myodes	glareolus	Male	adult	Yes	Yes	Yes	Yes	Yes	742	427	150	128	Am	NEGATIVE	NOT TESTE	0	Not Tested	Fleas
215	0	26/09/2019	D	25	Alive	11.5	Myodes	glareolus	Female	adult	Yes	Yes	Yes	Yes	Yes	753	333	170	124	Am	NEGATIVE	NOT TESTE	0	Not Tested	Flea, mites, talpa
214	0	26/09/2019	D	26	Alive	27.6	Apodemus	flavicolus	Male	adult	Yes	Yes	Yes	Yes	Yes	896	944	236	177	Am	NEGATIVE	NOT TESTE	0	Not Tested	Fleas and mites
213	0	26/09/2019	C	40	Dead	13.9	Myodes	glareolus	Male	adult	No	Yes	Yes	Yes	Yes	773	410	170	145	Am	POSITIVE	NEGATIVE	0	Not Tested	
212	0	26/09/2019	D	11	Alive	27.2	Apodemus	flavicolus	Male	adult	Yes	Yes	Yes	Yes	Yes	1010	974	240	174	Am	NEGATIVE	NOT TESTE	0	Not Tested	
211	1	26/09/2019	D	13	Alive	27.6	Apodemus	flavicolus	Male	adult	Yes	Yes	Yes	Yes	Yes	1015	1056	241	188	Am	POSITIVE	POSITIVE	512	NEGATIVE	
210	1	26/09/2019	D	6	Alive	15.7	Apodemus	agrarius	Female	adult	Yes	Yes	Yes	Yes	Yes	810	666	184	106	Am	POSITIVE	POSITIVE	128	POSITIVE	
209	1	26/09/2019	C	41	Dead	13.3	Myodes	glareolus	Male	juvenile	No	Yes	Yes	Yes	Yes	789	355	166	122	Am	POSITIVE	POSITIVE	8192	Not Tested	
208	0	26/09/2019	D	12	Dead	14.9	Apodemus	flavicolus	Male	juvenile	No	Yes	Yes	Yes	Yes	753	682	187	225	Am	NEGATIVE	NOT TESTE	0	Not Tested	
207	0	26/09/2019	D	8	Dead	46.3	Mustela	nivales	Female	adult	No	Yes	Yes	Yes	Yes	1590	436	226	92	Am	NOT TESTE	NEGATIVE	0	Not Tested	Weasel, mustela nivalis
206	0	26/09/2019	D	10	Alive	20	Myodes	glareolus	Male	juvenile	Yes	Yes	Yes	Yes	Yes	822	280	170	126	Am	NEGATIVE	NEGATIVE	0	Not Tested	used kidney for IFA test
205	0	26/09/2019	D	24	Alive	13	Apodemus	agrarius	Male	juvenile	Yes	Yes	Yes	Yes	Yes	770	653	183	123	Am	NEGATIVE	NOT TESTE	0	Not Tested	
204	0	26/09/2019	D	35	Dead	25.2	Apodemus	flavicolus	Female	adult	No	Yes	Yes	Yes	Yes	867	1041	236	195	Am	POSITIVE	NEGATIVE	0	NEGATIVE	
203	0	26/09/2019	D	14	Dead	31.5	Apodemus	flavicolus	Male	adult	No	Yes	Yes	Yes	Yes	882	979	257	170	Am	NEGATIVE	NOT TESTE	0	Not Tested	
202	1	26/09/2019	D	27	Dead	12.5	Microtus	arvalis	Male	adult	No	Yes	Yes	Yes	Yes	721	0	148	102	Am	NEGATIVE	NOT TESTE	512	Not Tested	No tail, tested IFA with kidney
201	1	26/09/2019	D	47	Alive	26.9	Apodemus	flavicolus	Female	adult	Yes	Yes	Yes	Yes	Yes	953	899	237	171	Am	POSITIVE	POSITIVE	8192	POSITIVE	
200	0	26/09/2019	D	4	Alive	18.3	Apodemus	flavicolus	Male	juvenile	Yes	Yes	Yes	Yes	Yes	826	900	234	152	Am	NEGATIVE	NOT TESTE	0	Not Tested	
199	0	26/09/2019	D	2	Dead	14.8	Apodemus	agrarius	Female	adult	No	Yes	Yes	Yes	Yes	721	694	176	115	Am	NEGATIVE	NOT TESTE	0	Not Tested	
198	0	26/09/2019	D	1	Alive	20.3	Apodemus	flavicolus	Male	juvenile	Yes	Yes	Yes	Yes	Yes	875	775	230	183	Am	NEGATIVE	NOT TESTE	0	Not Tested	
197	0	25/09/2019	B	43	Alive	18.5	Myodes	glareolus	Male	adult	Yes	Yes	Yes	Yes	Yes	935	365	135	127	Pm	NEGATIVE	NOT TESTE	0	Not Tested	
196	0	25/09/2019	B	24	Alive	15.7	Myodes	glareolus	Male	adult	Yes	Yes	Yes	Yes	Yes	861	361	156	132	Pm	NEGATIVE	NOT TESTE	0	Not Tested	
195	0	25/09/2019	A	9	Alive	15.1	Myodes	glareolus	Male	adult	Yes	Yes	Yes	Yes	Yes	856	358	159	142	Pm	NEGATIVE	NOT TESTE	0	Not Tested	
194	0	25/09/2019	E	33	Alive	18.7	Myodes	glareolus	Male	adult	Yes	Yes	Yes	Yes	Yes	892	355	152	146	Pm	NEGATIVE	NOT TESTE	0	Not Tested	
193	0	25/09/2019	D	48	Alive	16.1	Myodes	glareolus	Male	adult	Yes	Yes	Yes	Yes	Yes	874	336	168	144	Pm	NEGATIVE	NOT TESTE	0	Not Tested	
192	0	25/09/2019	D	31	Alive	22	Myodes	glareolus	Male	adult	Yes	Yes	Yes	Yes	Yes	950	445	173	133	Pm	POSITIVE	NEGATIVE	0	Not Tested	Fleas
191	0	25/09/2019	B	32	Alive	15.4	Myodes	glareolus	Male	juvenile	Yes	Yes	Yes	Yes	Yes	940	368	180	155	Pm	NEGATIVE	NOT TESTE	0	Not Tested	
190	0	25/09/2019	D	27	Alive	15	Microtus	arvalis	Male	juvenile	Yes	Yes	Yes	Yes	Yes	890	293	165	105	Pm	NEGATIVE	NOT TESTE	0	Not Tested	
189	0	25/09/2019	C	20	Alive	13.5	Myodes	glareolus	Male	adult	Yes	Yes	Yes	Yes	Yes	843	386	160	136	Pm	NEGATIVE	NOT TESTE	0	Not Tested	
188	0	25/09/2019	C	30	Alive	13.1	Myodes	glareolus	Male	adult	Yes	Yes	Yes	Yes	Yes	827	398	163	128	Pm	NEGATIVE	NOT TESTE	0	Not Tested	Fleas
187	0	25/09/2019	C	44	Alive	13.7	Myodes	glareolus	Male	adult	Yes	Yes	Yes	Yes	Yes	857	392	179	117	Pm	NEGATIVE	NOT TESTE	0	Not Tested	
186	0	25/09/2019	C	17	Alive	15	Myodes	glareolus	Male	adult	Yes	Yes	Yes	Yes	Yes	900	458	169	143	Pm	NEGATIVE	NOT TESTE	0	Not Tested	
185	0	25/09/2019	C	14	Alive	14.9	Myodes	glareolus	Male	adult	Yes	Yes	Yes	Yes	Yes	825	374	169	110	Pm	NEGATIVE	NOT TESTE	0	Not Tested	moribund
184	0	25/09/2019	C	24	Alive	21.6	Myodes	glareolus	Male	adult	Yes	Yes	Yes	Yes	Yes	924	454	153	138	Pm	NEGATIVE	NOT TESTE	0	Not Tested	
183	0	25/09/2019	D	14	Alive	23.4	Apodemus	flavicolus	Male	adult	Yes	Yes	Yes	Yes	Yes	848	910	229	165	Am	NEGATIVE	NOT TESTE	0	Not Tested	
182	0	25/09/2019	D	34	Alive	17.6	Myodes	glareolus	Male	adult	Yes	Yes	Yes	Yes	Yes	880	365	176	124	Am	NEGATIVE	NOT TESTE	0	Not Tested	
180	0	25/09/2019	D	26	Alive	25.7	Apodemus	flavicolus	Male	adult	Yes	Yes	Yes	Yes	Yes	960	962	235	176	Am	NEGATIVE	NOT TESTE	0	Not Tested	
179	0	25/09/2019	D	33	Alive	17.5	Myodes	glareolus	Male	adult	Yes	Yes	Yes	Yes	Yes	870	368	153	133	Am	NEGATIVE	NOT TESTE	0	Not Tested	Fleas
178	0	25/09/2019	C	16	Alive	13.8	Myodes	glareolus	Male	adult	Yes	Yes	Yes	Yes	Yes	827	360	152	122	Am	NEGATIVE	NOT TESTE	0	Not Tested	
177	0	25/09/2019	D	18	Dead	27.4	Apodemus	flavicolus	Female	adult	No	Yes	Yes	Yes	Yes	876	880	234	150	Am	NEGATIVE	NOT TESTE	0	Not Tested	
176	0	25/09/2019	D	17	Alive	16	Myodes	glareolus	Male	adult	Yes	Yes	Yes	Yes	Yes	896	380	164	124	Am	NEGATIVE	NOT TESTE	0	Not Tested	
175	0	25/09/2019	D	30	Dead	6.6	Sorex	minutus	Female	adult	No	Yes	Yes	Yes	Yes	632	398	131	179	Am	NEGATIVE	NOT TESTE	0	Not Tested	
174	1	25/09/2019	C	48	Dead	31.5	Apodemus	flavicolus	Male	adult	No	Yes	Yes	Yes	Yes	888	1020	232	157	Am	POSITIVE	NOT TESTE	128	POSITIVE	
173	0	25/09/2019	D	19	Alive	16.7	Myodes	glareolus	Male	adult	Yes	Yes	Yes	Yes	Yes	851	420	153	134	Am	NEGATIVE	NOT TESTE	0	Not Tested	
172	0	25/09/2019	C	6	Dead	26	Apodemus	flavicolus	Female	adult	No	Yes	Yes	Yes	Yes	897	980	233	165	Am	NEGATIVE	NOT TESTE	0	Not Tested	
171	0	25/09/2019	C	31	Dead	6.9	Sorex	minutus	Female	adult	No	Yes	Yes	Yes	Yes	656	334	124	57	Am	NEGATIVE	NOT TESTE	0	Not Tested	
170	0	25/09/2019	D	29	Alive	15.6	Myodes	glareolus	Male	adult	Yes	Yes	Yes	Yes	Yes	808	357	168	122	Am	NEGATIVE	NOT TESTE	0	Not Tested	
169	1	25/09/2019	D	8	Dead	21.6	Myodes	glareolus	Female	adult	No	Yes	Yes	Yes	Yes	866	428	165	135	Am	POSITIVE	POSITIVE	256	Not Tested	

168	0	25/09/2019	C	27	Dead	14.3	Apodemus	agrarius	Male	juvenile	No	Yes	Yes	Yes	Yes	767	680	186	115	Am	NEGATIVE	NOT TESTE	0	Not Tested	
167	0	25/09/2019	D	50	Alive	16.8	Myodes	glareolus	Male	adult	Yes	Yes	Yes	Yes	Yes	832	372	175	126	Am	NEGATIVE	NOT TESTE	0	Not Tested	
166	0	25/09/2019	D	11	Dead	15.4	Apodemus	agrarius	Male	adult	No	Yes	Yes	Yes	Yes	778	658	182	115	Am	NEGATIVE	NOT TESTE	0	Not Testec	Tail with infection
165	0	25/09/2019	C	11	Dead	15.3	Myodes	glareolus	Male	adult	No	Yes	Yes	Yes	Yes	813	328	178	127	Am	NEGATIVE	NOT TESTE	0	Not Tested	
164	0	25/09/2019	C	33	Alive	22	Myodes	glareolus	Male	adult	Yes	Yes	Yes	Yes	Yes	946	378	165	159	Am	NEGATIVE	NOT TESTE	0	Not Tested	
163	0	25/09/2019	C	2	Dead	15.8	Apodemus	agrarius	Male	adult	No	Yes	Yes	Yes	Yes	773	604	190	104	Am	NEGATIVE	NEGATIVE	0	Not Tested	
162	0	25/09/2019	D	13	Alive	18.8	Myodes	glareolus	Male	adult	Yes	Yes	Yes	Yes	Yes	875	360	174	130	Am	NEGATIVE	NOT TESTE	0	Not Tested	
161	1	25/09/2019	C	32	Dead	20.3	Myodes	glareolus	Male	adult	No	Yes	Yes	Yes	Yes	902	515	166	126	Am	POSITIVE	POSITIVE	1024	Not Tested	
160	1	25/09/2019	C	10	Alive	15.3	Apodemus	flavicolus	Male	juvenile	Yes	Yes	Yes	Yes	Yes	743	835	230	172	Am	POSITIVE	POSITIVE	256	POSITIVE	
159	0	25/09/2019	C	34	Dead	14.6	Myodes	glareolus	Male	adult	No	Yes	Yes	Yes	Yes	764	385	169	128	Am	NEGATIVE	NOT TESTE	0	Not Tested	
158	1	25/09/2019	C	35	Dead	19.1	Myodes	glareolus	Male	adult	No	Yes	Yes	Yes	Yes	854	426	155	126	Am	POSITIVE	POSITIVE	2048	Not Tested	
157	1	25/09/2019	C	36	Alive	23.2	Apodemus	flavicolus	Male	adult	Yes	Yes	Yes	Yes	Yes	960	957	252	169	Am	POSITIVE	POSITIVE	128	NEGATIVE	
156	1	25/09/2019	C	40	Dead	12.4	Myodes	glareolus	Male	juvenile	No	Yes	Yes	Yes	Yes	747	349	175	133	Am	POSITIVE	POSITIVE	256	Not Tested	
155	0	25/09/2019	C	9	Alive	15.2	Myodes	glareolus	Male	adult	Yes	Yes	Yes	Yes	Yes	848	397	167	124	Am	NEGATIVE	NOT TESTE	0	Not Tested	
154	0	25/09/2019	C	23	Dead	13.3	Myodes	glareolus	Male	juvenile	No	Yes	Yes	Yes	Yes	746	357	163	135	Am	POSITIVE	NEGATIVE	0	Not Testec	Larva ticks
153	0	25/09/2019	C	19	Alive	20.9	Myodes	glareolus	Male	adult	Yes	Yes	Yes	Yes	Yes	910	409	165	138	Am	NEGATIVE	NOT TESTE	0	Not Tested	
152	0	25/09/2019	D	37	Dead	26.4	Apodemus	flavicolus	Male	adult	No	Yes	Yes	Yes	Yes	844	990	244	167	Am	NEGATIVE	NOT TESTE	0	Not Tested	
151	0	25/09/2019	C	5	Alive	29	Apodemus	flavicolus	Male	adult	Yes	Yes	Yes	Yes	Yes	996	1097	223	173	Am	NEGATIVE	NOT TESTE	0	Not Tested	
150	0	25/09/2019	C	37	Dead	14.5	Myodes	glareolus	Female	adult	No	Yes	Yes	Yes	Yes	784	371	170	125	Am	NEGATIVE	NOT TESTE	0	Not Tested	
149	0	25/09/2019	D	38	Alive	15.2	Myodes	glareolus	Female	adult	Yes	Yes	Yes	Yes	Yes	879	418	160	138	Am	NEGATIVE	NOT TESTE	0	Not Tested	
148	0	25/09/2019	D	39	Dead	13.7	Myodes	glareolus	Female	adult	No	Yes	Yes	Yes	Yes	757	418	148	130	Am	NEGATIVE	NOT TESTE	0	Not Tested	
147	0	25/09/2019	D	49	Alive	26.6	Apodemus	flavicolus	Female	adult	Yes	Yes	Yes	Yes	Yes	1031	955	221	167	Am	NEGATIVE	NOT TESTE	0	Not Testec	Fleas
146	0	25/09/2019	D	35	Dead	12.4	Myodes	glareolus	Male	juvenile	No	Yes	Yes	Yes	Yes	735	345	150	112	Am	NEGATIVE	NOT TESTE	0	Not Tested	
145	0	25/09/2019	D	35	Alive	13.2	Myodes	glareolus	Male	juvenile	Yes	Yes	Yes	Yes	Yes	883	346	145	106	Am	POSITIVE	NEGATIVE	0	Not Testec	Moribund
144	0	25/09/2019	D	14	Dead	14	Myodes	glareolus	Male	adult	No	Yes	Yes	Yes	Yes	826	393	150	112	Am	NEGATIVE	NOT TESTE	0	Not Tested	
143	0	25/09/2019	D	16	Alive	24.3	Apodemus	flavicolus	Male	adult	Yes	Yes	Yes	Yes	Yes	958	914	232	124	Am	NEGATIVE	NOT TESTE	0	Not Tested	
142	1	25/09/2019	D	3	Dead	15	Myodes	glareolus	Female	adult	No	Yes	Yes	Yes	Yes	878	454	164	157	Am	POSITIVE	POSITIVE	256	Not Tested	
141	0	25/09/2019	D	12	Alive	21.5	Apodemus	flavicolus	Male	juvenile	Yes	Yes	Yes	Yes	Yes	921	896	205	160	Am	NEGATIVE	NOT TESTE	0	Not Tested	
140	0	25/09/2019	D	2	Dead	26.8	Apodemus	flavicolus	Female	adult	No	Yes	Yes	Yes	Yes	942	996	203	155	Am	NEGATIVE	NOT TESTE	0	Not Tested	
139	1	25/09/2019	D	7	Alive	20.6	Apodemus	flavicolus	Male	juvenile	Yes	Yes	Yes	Yes	Yes	887	986	216	170	Am	POSITIVE	POSITIVE	64	NEGATIVE	
138	1	25/09/2019	D	6	Dead	15.2	Apodemus	agrarius	Male	adult	No	Yes	Yes	Yes	Yes	741	740	195	122	Am	POSITIVE	POSITIVE	1024	POSITIVE	
137	0	25/09/2019	B	41	Alive	17.5	Myodes	glareolus	Female	adult	Yes	Yes	Yes	Yes	Yes	834	395	145	138	Am	NEGATIVE	NOT TESTE	0	Not Tested	
136	0	25/09/2019	D	1	Dead	17.5	Apodemus	agrarius	Male	adult	No	Yes	Yes	Yes	Yes	860	714	194	101	Am	NEGATIVE	NEGATIVE	0	Not Testec	Tail with infection
135	0	25/09/2019	B	28	Alive	33.6	Apodemus	flavicolus	Male	adult	Yes	Yes	Yes	Yes	Yes	1130	1046	216	194	Am	NEGATIVE	NOT TESTE	0	Not Testec	Fleas
134	0	25/09/2019	B	47	Dead	40.4	Myodes	glareolus	Male	adult	No	Yes	Yes	Yes	Yes	852	305	155	120	Am	NEGATIVE	NOT TESTE	0	Not Tested	
133	0	25/09/2019	B	17	Alive	41.7	Apodemus	flavicolus	Male	adult	Yes	Yes	Yes	Yes	Yes	1131	1062	252	190	Am	NEGATIVE	NOT TESTE	0	Not Tested	
132	0	25/09/2019	B	32	Dead	12.1	Myodes	glareolus	Female	adult	No	Yes	Yes	Yes	Yes	780	410	238	135	Am	POSITIVE	NEGATIVE	0	Not Tested	
131	0	25/09/2019	B	33	Alive	16.3	Myodes	glareolus	Male	adult	Yes	Yes	Yes	Yes	Yes	879	425	145	99	Am	NEGATIVE	NOT TESTE	0	Not Tested	
130	0	25/09/2019	B	24	Dead	13.2	Myodes	glareolus	Male	adult	No	Yes	Yes	Yes	Yes	745	415	138	116	Am	NEGATIVE	NOT TESTE	0	Not Tested	
129	0	25/09/2019	D	46	Alive	25.2	Apodemus	flavicolus	Female	adult	Yes	Yes	Yes	Yes	Yes	912	109	225	165	Am	NEGATIVE	NOT TESTE	0	Not Tested	
128	0	25/09/2019	B	22	Alive	15.4	Myodes	glareolus	Female	adult	Yes	Yes	Yes	Yes	Yes	878	334	132	114	Am	NEGATIVE	NOT TESTE	0	Not Tested	
127	0	25/09/2019	A	7	Dead	23.2	Apodemus	flavicolus	Female	adult	No	Yes	Yes	Yes	Yes	870	942	236	165	Am	NEGATIVE	NOT TESTE	0	Not Tested	
126	1	25/09/2019	A	2	Dead	16.9	Myodes	glareolus	Male	adult	No	Yes	Yes	Yes	Yes	884	393	143	94	Am	POSITIVE	POSITIVE	4096	Not Tested	
125	1	25/09/2019	A	12	Alive	37.5	Apodemus	flavicolus	Male	adult	Yes	Yes	Yes	Yes	Yes	1072	0	228	183	Am	POSITIVE	POSITIVE	512	NEGATIVE	Cut tail, missing age (assumed adult)
124	0	25/09/2019	A	8	Dead	7.1	Sorex	minutus	Female	adult	No	Yes	Yes	Yes	Yes	625	267	98	54	Am	NEGATIVE	NOT TESTE	0	Not Testec	Missing age (assumed to be adult)
123	0	25/09/2019	A	42	Alive	12.8	Microtus	subterrane	Female	adult	Yes	Yes	Yes	Yes	Yes	774	250	114	98	Am	NEGATIVE	NOT TESTE	0	Not Testec	Microtus subterranus
122	0	24/09/2019	B	24	Alive	15.4	Myodes	glareolus	Male	juvenile	Yes	Yes	Yes	Yes	Yes	804	350	175	123	Pm	NEGATIVE	NOT TESTE	0	Not Testec	Mites
121	0	24/09/2019	B	37	Alive	17.5	Myodes	glareolus	Male	juvenile	Yes	Yes	Yes	Yes	Yes	842	394	124	149	Am	NEGATIVE	NOT TESTE	0	Not Tested	
120	0	24/09/2019	A	18	Alive	5.5	Sorex	minutus	Male	adult	Yes	Yes	Yes	Yes	Yes	604	349	117	44	Am	NEGATIVE	NOT TESTE	0	Not Tested	
119	0	24/09/2019	A	7	Dead	29.1	Apodemus	flavicolus	Male	adult	No	Yes	Yes	Yes	Yes	923	1106	238	193	Am	POSITIVE	NEGATIVE	0	NEGATIVE	
118	0	24/09/2019	A	9	Dead	23.4	Apodemus	flavicolus	Male	adult	No	Yes	Yes	Yes	Yes	911	955	233	186	Am	NEGATIVE	NOT TESTE	64	NEGATIVE	Mites (gamasid), used kidney for titer
117	0	24/09/2019	B	39	Dead	13.8	Myodes	glareolus	Male	juvenile	No	Yes	Yes	Yes	Yes	786	327	173	140	Am	NEGATIVE	NOT TESTE	0	Not Tested	
116	0	24/09/2019	B	11	Dead	12.4	Myodes	glareolus	Male	juvenile	No	Yes	Yes	Yes	Yes	736	320	145	138	Am	NEGATIVE	NOT TESTE	0	Not Tested	
115	0	24/09/2019	B	29	Dead	33.5	Apodemus	flavicolus	Male	juvenile	No	Yes	Yes	Yes	Yes	954	960	219	186	Am	NEGATIVE	NOT TESTE	0	Not Testec	Mites
114	0	24/09/2019	B	7	Dead	15.2	Myodes	glareolus	Male	juvenile	No	Yes	Yes	Yes	Yes	810	359	147	139	Am	POSITIVE	NOT TESTE	128	Not Tested	
113	0	24/09/2019	B	17	Dead	15	Myodes	glareolus	Male	juvenile	No	Yes	Yes	Yes	Yes	755	394	153	128	Am	NEGATIVE	NOT TESTE	0	Not Tested	
112	0	24/09/2019	A	17	Dead	23.2	Apodemus	flavicolus	Female	juvenile	No	Yes	Yes	Yes	Yes	863	1005	223	169	Am	NEGATIVE	NOT TESTE	0	Not Tested	
111	0	24/09/2019	B	14	Dead	32.7	Apodemus	flavicolus	Female	adult	No	Yes	Yes	Yes	Yes	963	992	226	186	Am	NEGATIVE	NOT TESTE	0	Not Tested	
110	0	24/09/2019	B	13	Dead	34.6	Apodemus	flavicolus	Female	juvenile	No	Yes	Yes	Yes	Yes	926	1115	225	187	Am	NEGATIVE	NOT TESTE	0	Not Tested	
109	0	24/09/2019	B	9	Alive	26.4	Apodemus	flavicolus	Male	juvenile	Yes	Yes	Yes	Yes	Yes	1028	947	244	170	Am	NEGATIVE	NOT TESTE	0	Not Tested	
108	0	24/09/2019	B	23	Dead	14.5	Myodes	glareolus	Male	juvenile	No	Yes	Yes	Yes	Yes	710	340	166	150	Am	NEGATIVE	NOT TESTE	0	Not Testec	Ectoparasites
107	0	24/09/2019	B	8	Alive	16.2	Myodes	glareolus	Male	juvenile	Yes	Yes	Yes	Yes	Yes	870	458	145	153	Am	POSITIVE	NEGATIVE	0	Not Tested	
106	0	24/09/2019	A	5	Dead	32.2	Apodemus	flavicolus	Female	adult	No	Yes	Yes	Yes	Yes	936	1003	237	183	Am	NEGATIVE	NOT TESTE	0	Not Tested	
105	0	24/09/2019	A	46	Alive	22.2	Apodemus	flavicolus	Male	juvenile	Yes	Yes	Yes	Yes	Yes	940	862	243	152	Am	NEGATIVE	NOT TESTE	0	Not Tested	
104	0	24/09/2019	A	49	Dead	14.1	Myodes	glareolus	Male	juvenile	No	Yes	Yes	Yes	Yes	746	424	125	126	Am	NEGATIVE	NOT TESTE	0	Not Tested	
103	0	24/09/2019	A	34	Alive	30.6	Apodemus	flavicolus	Male	adult	Yes	Yes	Yes	Yes	Yes	1056	1042	249	157	Am	NEGATIVE	NOT TESTE	0	Not Tested	
102	0	24/09/2019	A	40	Alive	16.2	Myodes	glareolus	Male	juvenile	Yes	Yes	Yes	Yes	Yes	875	374	137	92	Am	NEGATIVE	NOT TESTE	0	Not Tested	
101	0	24/09/2019	B	25	Alive	18	Myodes	glareolus	Male	adult	Yes	Yes	Yes	Yes	Yes	925	424	138	135	Am	NEGATIVE	NOT TESTE	0	Not Tested	
181	0	25/09/2019	D	42	Alive	15.3	Myodes	glareolus	Male	adult	Yes	Yes	Yes	Yes	Yes	835	348	159	112	Am	NEGATIVE	NOT TESTE	0	Not Tested	
100	0	24/09/2019	B	33	Alive	38.6	Apodemus	flavicolus	Male	juvenile	Yes	Yes	Yes	Yes	Yes	1143	1150	243	193	Am	NEGATIVE	NOT TESTE	0	Not Testec	none
