# Supplementary material for: Prevalence of Hantaviruses Harbored by Murid Rodents in Northwestern Ukraine and Discovery of a Novel Puumala Virus Strain
Source: Viruses. 2021 Aug 18;13(8):1640. doi: 10.3390/v13081640 (PMC8402871; doi:10.3390/v13081640)
Supplement: Supplementary file 1 [file viruses-13-01640-s001.zip › TableS4 Primers used to amplify DOBV or PUUV hantaviruses.pdf]

Table S4. Primers used to amplify DOBV or PUUV hantaviruses. DOBV primers were designed based on an alignment of sequences from Russia, Germany, Slovenia, Slovakia, Croatia, Estonia, Greece, Finland from Apodemus agrarius and A. flavicollis rodents and Homo sapiens hosts. PUUV primers were designed based on alignments from sequences collected from Russia, Germany, Slovenia, Slovakia, Finland, France, Czech Republic. The optimal annealing temperature for these primers is set at 60°C.

PUUV Small Segment Primers

No.	Forward Primer	Forward Primer Sequence	Reverse Primer	Reverse Primer Sequence
1	1-30F	TAG TAG TAG ACT CCT TGA AAA GCT ACT ACG	538-563R	GGTCTYCTRATRCCATTRATRTCCTC
2			958-980R	GCAAAKACCCAWGGKGCATTAGG
3	382-404F	GGYCARACAGCAGAYTGGTATAC	1891-1920R	TAGTAGTAGACTCCTTGAAAAGCAATCAAG
4	883-910F	CATGTGGCWGAYATYGATAAGTTRATTG		

PUUV Medium Segment Primers

No.	Forward Primer	Forward Primer Sequence	Reverse Primer	Reverse Primer Sequence
1	1-26F	TAG TAG TAG ACT CCG CAA GAA GAA GC	499-525R	GTTGTTATRCAWGTYTGDATAGGTCCC
2			999-1023R	CCTGAAAATGCAATMCCYTGTACTG
3	467-490F	CARACAGYRTGTCARCCTACTGTC	1691-1715R	GACASACTTCACAWACCATKGARCC
4	990-1014F	GAATCATCWGAYACAGTACARGGKATWGC	2040-2061R	GATCCATGTGCTGTRTCWGTCC
5	1691-1715F	GGYTCMATGGTWTGTGAAGTSTGTC	2754-2774R	GTYTGYTCWGTRCCMARYTGAATGC
6	2040-2061F	GGACWGAYACAGCACATGGATC	3092-3117R	CATAACACATWGCWGMRTCACAKGC
7	2397-2419F	GGMTGTACWGCWTGTGGTGTWTAY	3656-3682R	TAG TAG TAG RCT CCG CAR CAA CAA AAG
8	2997-3021F	CWATWGATGGGGCATGGGGTTC		

PUUV Large Segment Primers

No.	Forward Primer	Forward Primer Sequence	Reverse Primer	Reverse Primer Sequence
1	1-26F	TAG TAG TAG ACT CCG AGA TAG AGA AR	542-563R	CCATCWGTYCTWAYWGCYACCAC
2			1170-1198R	CATCAATTARTGCYTTTGAYTTWGGATCC
3	542-563F	GTGGTRGCWRTWAGRACWGATGG	1498-1520R	GATCTYTTAAGCCCTGCATGTGC
4	871-898F	GCATCMAARTATGGACAYTCWAGAAAGC	1927-1952R	CCAGARTAKGTAGATGTRACWGCTGG
5	1498-1520F	GCACATGCAGGGCTTAARAGATC	2542-2567R	GCTAAATGACCATCTTCYTGTAATGC
6	1927-1952F	CCAGCWGTYACATCTACMTAYTCTGG	3047-3076R	CATAWATRTTYCTTARGGCATCAACTACAC
7	2542-2567F	GCATTACARGAAGATGGTCATTTAGC	3429-3453R	CCACATYTCYTGATTMACAGCATGC
8	3047-3076F	GTG TAG TTG ATG CCY TAA GRA AYA TWT ATG	4036-4061R	GTRAAYACTTTCCATTGWACTTTCCC
9	3429-3453F	GCATGCTGTKAATCARGARATGTGG	4423-4450R	GTACTTCATTYARRAARTCTCTCCAKGC
10	4036-4061F	GGGAAAGTWCAATGGAAAGTRTTYAC	4915-4939R	CYTCRATRCATARTCTCATTGCAGC
11	4423-4450F	GCMTGGAGAGAYTTYYTRAATGAAGTAC	5568-5591R	GGATTYTCTTCATCWAGYTGTGCC
12	4915-4939F	GCTGCAATGAGAYTATGYATYGARG	6064-6088R	CTCCAGTRTAAGCWGAYTCATCYTG
13	5568-5591F	GGCACARCTWGATGAAGARAATCC	6527-6550R	GA GAA AAG AGC CTC GTA TGA TGA T
14	6064-6088F	CARGATGARTCWGCTTAYACTGGAG		

Reference sequences included: S-segment (M32750, KT885052, L08804, JQ319168, JQ319171, KY364995, KT247592, JN831943, JN657232, FN377821, KY364998, KT247597, KY365001, KT247594, KY364996), M-segment (KT885051, L08754, HE801634, JQ319175, KT247602, KT247600, KJ994777, MF077609, MF077578, MF077580, MF077574, AJ888741, AJ888742), L-segment (KT885050, EF405801, NC_005225, KT247608, KT247606, Z66548, JN831945, KJ994778, KR047483, MF077569, MF077567, KF776674, KF776670)

DOBV Small Segment Primers

No.	Forward Primer	Forward Primer Sequence	Reverse Primer	Reverse Primer Sequence
1	1-30F	TAGTAGTAGRCTCCCTAAARAGCACTAYAC	572-595R	CTR GAY TGD GCA TTR GGC ATT GAC
2			988-1011R	CCATGCCTGCAATRAACAGGCAGG
3	515-539F	GGA TGA CAG CTC DTT YGA RGA TGT G	1687-1721R	TAGTAGTAGRCTCCCTAAAAAGACATTCAGGAAGC
4	1033-1057F	CTG TCC TYC AGG AYA TGA GGA AYA C		

DOBV Medium Segment Primers

No.	Forward Primer	Forward Primer Sequence	Reverse Primer	Reverse Primer Sequence
1	1-25F	TAG TAG TAG ACT CCG CAA GAA AYA G	584-608R	GGTCTGGYACAAARCAYTTCCCTTC
2			1139-1164R	GGA CCG ATR ATA CTT TGT CAR GTG GG
3	457-480F	GCC AAC GAT GCA TAT GAT TGT KCC	1496-1520R	C AAA GCA GAA TGT RGT GAG RAG AGC
4	962-984F	GCA GCA AAY ATG CTV YTY ACT GC	2010-2033R	CAA CAC CAT GTG CRT TGT CAT TCC
5	1430-1452F	CCT GGK GTA GCA CAT TCW ATT GC	2549-2574R	GGT ATC ACC TTG TGA GAA CTT TGA AAC
6	1860-1881	CAC CTC ART CWA CAA GYC CDG G	3212-3233R	C YAR RTG YGG WGC RCT WGC TTG
7	2438-2465	GTY CGT TAY AGY MGB AAA GTN TGT GTY C	3620-3644R	TAGTAGTAKRCTCCGCARGATATAG
8	2873-2899F	GATGGRATGCTTAARGACCAYHTCAAY		

DOBV Large Segment Primers

No.	Forward Primer	Forward Primer Sequence	Reverse Primer	Reverse Primer Sequence
1	1-30F	TAGTAGTAGACTCCGGAARAGACAARYTAC	569-593R	G CCT CAT GTA TTG AAC AAC ACC ATC
2			1097-1119R	GG TAT GAA YTG GGT TTT GGG TGC
3	412-439F	GCT TAA GTA TGA GGC AGG RYT AAA GTAC	1598-1625R	C TGT YAC AAA CCT TAT ATA AGA CCC TGC
4	950-974F	GCT ACA ATY CTT TCT GAY CAR GTH G	2118-2140R	GT AAA TCC CAC TTG CWC CRA CAG
5	1452-1476F	CAT GGC ATM TTG GGC AYC TYA TTM G	2567-2592R	C TAC AGC AGC AAG TTC TAC AGC ATC
6	1928-1953F	CCT GCT GTY ACA TCW CTT TAC TCT GG	3228-3253R	CATYCCKACACCRAAAAGAGATGAAC
7	2536-2559F	GTC AGG HCA RGT TCA RGA AGA TGG	3566-3589R	KGG KAT TGA YAC AGC ACA DCY TTC
8	2948-2970F	GCT GAT GCA ACR AAA TGG TCT CC	4218-4241R	GKG GTG AYT GCA TTG TCA TRG CAG
9	3566-3589F	GAA RGH TGT GCT GTR TCA ATM CCM	4694-4719R	GG TGC AAC YTT YTT CAT YAC YTC AGC
10	4037-4062F	CAR CAT GAA AGR YTR GGY GAR TTY TC	5096-5118R	GC AGC ACA YTG RAT YTC TGG RTC
11	4571-4598F	GCAGTVACAGTTGATGAAATGTCAGATG	5750-5769R	GC AGG AGG TAT TGC WGC TGG
12	5079-5106F	GTGCTGCAYTRATGATWGCAGATRTMAG	6327-6353R	CRA ATG TRS TGA ACT GAT CAA CAA CAC
13	5603-5630F	CTWGCACATGCWTTTCATACWATMAGRG	6503-6535R	TAGTAGTAGTATGCTCCGGAAAATGAAAATRAA
14	5951-5978F	GTY GAT GAR GAT GAA GAG RTT GATGATG		

Reference sequences included: S-segment (JQ026204, GU904032, GU904031, GU904030, GU904029, GU904028, GU904027, GQ205407, GQ205408, GQ205406, GQ205405, GQ205404, GQ205403, GQ205402, GQ205401, KT885043, KC676608), M-segment (AY961616, AY168578, AY168577, NC_005234, DL139689, DI163052, AJ410616, AJ009774, HV984406, L33685, MH251335, KT885042, JQ026205, JF920149, GU904038, GU904037, GU904036, GU904035, GU904034, GU904033, GQ205413, GQ205412, GQ205411, GQ205410, GQ205409, EU188453, EU188450), L-segment (NC_005235, JQ026206, MH251336, AJ410617, KT885041, JF920148, GU904044, GU904045, GU904043, GU904042, GU904041, GU904040, EU188450)
