# Supplementary material for: Prevalence of Hantaviruses Harbored by Murid Rodents in Northwestern Ukraine and Discovery of a Novel Puumala Virus Strain
Source: Viruses. 2021 Aug 18;13(8):1640. doi: 10.3390/v13081640 (PMC8402871; doi:10.3390/v13081640)
Supplement: Supplementary file 1 [file viruses-13-01640-s001.zip › TableS5 NGS depth of coverage.pdf]

Table S5. NGS depth of coverage of conserved S-, M-, L-segment sites in Ukraine isolates from sequenced vRNA. * represents sites of low coverage that did not have enough depth of coverage to report. M.g. (*Myodes glareolus*)

	Puumala virus Gene	S segment		M segment				L segment				
	Amino acid subsitution	N168K	Q428H	Q/R/I/M57K	G/T/S78A	S185A	N/T244S	L759F	S761G	Q/R/H777L	S1646A	D2140E
Animal ID No.	M.g. 274	13305X	45X	538X	1174-1429X	14X	6X	24X	24X	58X	*	*
	M.g. 272	7x	*	6X	2-4X	*	*	*	*	39X	*	*
	M.g. 209	4222X	1080X	62X	132-142X	32X	47X	5X	5X	1045X	13X	1X
	M.g. 252	5X	*	4X	5-6X	*	*	9X	9X	116X	*	*
	M.g. 394	2373X	112X	1X	173-213X	159X	647X	1463X	1458X	169X	2X	*
	M.g. 156	10168X	4425X	480X	1264-1422X	372X	391X	28X	28X	4376X	35X	21X
	M.g. 256	16241X	1672X	689X	1418X	264X	6X	29X	29X	9028X	38X	7X
	M.g. 258	613X	290X	4X	9-11X	4X	*	15X	15X	310X	*	*
	M.g. 400	473X	66X	*	21-23X	29X	145X	398X	398X	24X	*	*
	M.g. 355	358X	17X	*	17-22X	24X	3X	111X	111X	21X	*	*
	M.g. 412	1980X	3X	*	84-95X	50X	285X	821X	822X	2X	*	*
	M.g. 321	523X	*	*	40-47X	*	10X	10997X	10994X	49X	*	*
	M.g. 416	6581X	*	*	166-222X	983X	3357X	3434X	3432X	19X	*	3X
	M.g. 243	59X	*	1X	2-4X	*	2X	5X	5X	318X	*	*
	M.g. 338	2X	1X	*	1X	*	*	25X	25X	10X	*	*
	M.g. 169	6366X	1551X	237X	540-650X	106X	46X	9X	9X	1627X	3X	*
	M.g. 158	1X	1X	28X	47-55X	*	*	*	*	17X	*	*
	M.g. 142	2796X	*	27X	64-80X	*	*	2X	2X	884X	*	*
